# Immunogenicity and efficacy of the COVID-19 candidate vector vaccine MVA-SARS-2-S in preclinical vaccination

**DOI:** 10.1073/pnas.2026207118

**Published:** 2021-06-23

**Authors:** Alina Tscherne, Jan Hendrik Schwarz, Cornelius Rohde, Alexandra Kupke, Georgia Kalodimou, Leonard Limpinsel, Nisreen M. A. Okba, Berislav Bošnjak, Inga Sandrock, Ivan Odak, Sandro Halwe, Lucie Sauerhering, Katrin Brosinski, Nan Liangliang, Elke Duell, Sylvia Jany, Astrid Freudenstein, Jörg Schmidt, Anke Werner, Michelle Gellhorn Serra, Michael Klüver, Wolfgang Guggemos, Michael Seilmaier, Clemens-Martin Wendtner, Reinhold Förster, Bart L. Haagmans, Stephan Becker, Gerd Sutter, Asisa Volz

**Affiliations:** ^a^Division of Virology, Department of Veterinary Sciences, Ludwig-Maximilians-Universität München, 80539 Munich, Germany;; ^b^Division of Virology, Department of Veterinary Sciences, German Center for Infection Research, 80539 Munich, Germany;; ^c^Institute of Virology, Philipps University Marburg, 35037 Marburg, Germany;; ^d^Institute of Virology, Marburg, German Center for Infection Research, 35392 Giessen-Marburg-Langen, Germany;; ^e^Department of Viroscience, Erasmus Medical Center, 3015 CN Rotterdam, The Netherlands;; ^f^Institute of Immunology, Hannover Medical School, 30625 Hannover, Germany;; ^g^Munich Clinic Schwabing, Academic Teaching Hospital, Ludwig-Maximilians-Universität München, 80804 Munich, Germany;; ^h^Institute of Immunology, German Center for Infection Research, 30625 Hannover, Germany;; ^i^Cluster of Excellence RESIST (EXC 2155), Hannover Medical School, 30625 Hannover, Germany;; ^j^Institute of Virology, University of Veterinary Medicine, Hannover, 30559 Hannover, Germany

**Keywords:** vaccine vector, vaccinia virus, poxvirus, nonclinical testing

## Abstract

Severe acute respiratory syndrome (SARS) coronavirus 2 (SARS-CoV-2) has emerged as the infectious agent causing the pandemic coronavirus disease 2019 (COVID-19) with dramatic consequences for global human health and economics. Previously, we reached clinical evaluation with our vector vaccine based on modified vaccinia virus Ankara (MVA) against the Middle East respiratory syndrome coronavirus (MERS-CoV), which causes an infection in humans similar to SARS and COVID-19. Here, we describe the construction and preclinical characterization of a recombinant MVA expressing full-length SARS-CoV-2 spike (S) protein (MVA-SARS-2-S). Genetic stability and growth characteristics of MVA-SARS-2-S, plus its robust expression of S protein as antigen, make it a suitable candidate vaccine for industrial-scale production. Vaccinated mice produced S-specific CD8^+^ T cells and serum antibodies binding to S protein that neutralized SARS-CoV-2. Prime-boost vaccination with MVA-SARS-2-S protected mice sensitized with a human ACE2-expressing adenovirus from SARS-CoV-2 infection. MVA-SARS-2-S is currently being investigated in a phase I clinical trial as aspirant for developing a safe and efficacious vaccine against COVID-19.

## Significance

The highly attenuated vaccinia virus MVA is licensed as smallpox vaccine; as a vector it is a component of the approved adenovirus-MVA–based prime-boost vaccine against Ebola virus disease. Here, we provide results from testing the COVID-19 candidate vaccine MVA-SARS-2-S, a poxvirus-based vector vaccine that proceeded to clinical evaluation. When administered by intramuscular inoculation, MVA-SARS-2-S expresses and safely delivers the full-length SARS-CoV-2 S protein, inducing balanced SARS-CoV-2–specific cellular and humoral immunity, and protective efficacy in vaccinated mice. Substantial clinical experience has been gained with MVA vectors using homologous and heterologous prime-boost applications, including the immunization of children and immunocompromised individuals. Thus, MVA-SARS-2-S represents an important resource for developing further optimized COVID-19 vaccines.

Severe acute respiratory syndrome coronavirus 2 (SARS-CoV-2), the causal agent of coronavirus disease 2019 (COVID-19), first emerged in late 2019 in China (1). SARS-CoV-2 exhibits extremely efficient human-to-human transmission, the new pathogen rapidly spread worldwide, and within months it caused a global pandemic, changing daily life for billions of people. The COVID-19 case fatality rate of ∼2–5% makes the development of countermeasures a global priority. In fact, the development of COVID-19 vaccine candidates is advancing at an international level with unprecedented speed. About 1 y after the first known cases of COVID-19, we can account for >80 SARS-CoV-2–specific vaccines in clinical evaluations and >10 candidate vaccines already in phase III trials (2–4). However, we still lack information on the key immune mechanisms needed for protection against COVID-19. A better understanding of the types of immune response elicited upon natural SARS-CoV-2 infections has become an essential component to assess the promise of various vaccination strategies (5).

The SARS-CoV-2 spike (S) protein serves as the most important target antigen for vaccine development based on preclinical research on candidate vaccines against SARS-CoV or Middle East respiratory syndrome coronavirus (MERS-CoV). The trimeric S protein is a prominent structure at the virion surface and essential for SARS-CoV-2 cell entry. As a class I viral fusion protein, it mediates virus interaction with the cellular receptor angiotensin-converting enzyme 2 (ACE2), and fusion with the host cell membrane, both key steps in infection. Thus, infection can be prevented by S-specific antibodies neutralizing the virus (6–9).

Among the front-runner vaccines are new technologies such as messenger RNA (mRNA)-based vaccines and nonreplicating adenovirus vector vaccines (10–13). First reports from these SARS-CoV-2-S–specific vaccines in phase 1/2 clinical studies demonstrated acceptable safety and promising immunogenicity profiles, and by now data from large phase 3 clinical trials show promising levels of protective efficacy (4, 12–14). In December 2020, the first mRNA-based COVID-19 vaccines received emergency use authorization or conditional licensing by the US Food and Drug Administration and European Medicines Agency (11, 15, 16). By March 2021, two adenovirus vector-based COVID-10 vaccines had been approved by regulatory authorities (17, 18). This is good news because efficacious vaccines will provide a strategy to change SARS-CoV-2 transmission dynamics. In addition, multiple vaccine types will be advantageous to meet specific demands across different target populations. This includes the possibility of using heterologous immunization strategies depending on an individual’s health status, boosting capacities, and the need for balanced humoral and Th1-directed cellular immune responses.

MVA, a highly attenuated strain of vaccinia virus originating from growth selection on chicken embryo tissue cultures, shows a characteristic replication defect in mammalian cells but allows unimpaired production of heterologous proteins (19). At present, MVA serves as an advanced vaccine technology platform for developing new vector vaccines against infectious disease including emerging viruses and cancer (20). In response to the ongoing pandemic, the MVA vector vaccine platform allows rapid generation of experimental SARS-CoV-2–specific vaccines (21). Previous work from our laboratory addressed the development of an MVA candidate vaccine against MERS with immunizations in animal models demonstrating the safety, immunogenicity, and protective efficacy of MVA-induced MERS-CoV S-antigen–specific immunity (22–25). Clinical safety and immunogenicity of the MVA-MERS-S candidate vaccine was established in a first-in-human phase I clinical study under funding from the German Center for Infection Research (DZIF) (26).

Here, we show that a recombinant MVA produces the full-length S protein of SARS-CoV-2 as ∼190- to 200-kDa N-glycosylated protein. Our studies confirmed cleavage of the mature full-length S protein into an amino-terminal domain (S1) and a ∼80- to 100-kDa carboxyl-terminal domain (S2) that is anchored to the membrane. When tested as a vaccine in BALB/c mice, recombinant MVA expressing the S protein induced SARS-CoV-2–specific T cells and antibodies, and robustly protected vaccinated animals against lung infection upon SARS-CoV-2 challenge.

## Results

### Design and Generation of Candidate MVA Vector Viruses.

**Fig. 1.**
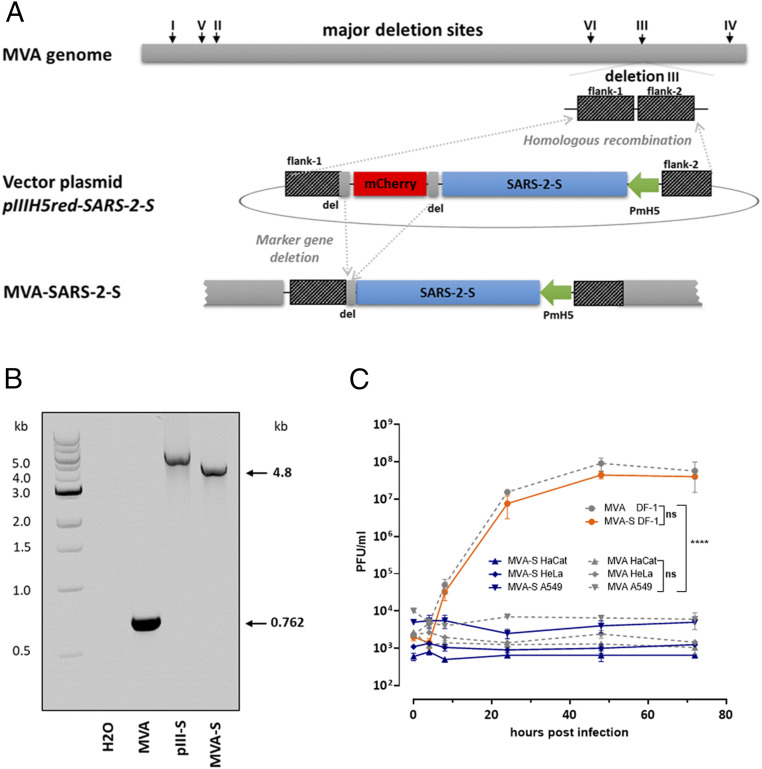
Construction and virological characterization of MVA-SARS-2-S. (*A*) Schematic diagram of the MVA genome with the major deletion sites I to VI. The site of deletion III served for insertion of the SARS-CoV-2 S gene sequence (SARS-2-S). SARS-2-S was controlled by the virus-specific promoter PmH5 and inserted via homologous recombination between MVA DNA sequences (flank-1 and flank-2) adjacent to deletion site III in the MVA genome and copies cloned in the MVA vector plasmid pIIIH5red-SARS-2-S. Expression of the red fluorescent marker protein mCherry was used during plaque purification. Repetition of short flank-1 derived DNA sequences (del) served to remove the marker gene by intragenomic homologous recombination (marker gene deletion). (*B*) Genetic integrity of MVA-SARS-2-S (MVA-S). PCR analysis of viral DNA with deletion III site-specific oligonucleotide primers confirmed insertion of the SARS-2-S sequence and intragenomic deletion of the marker gene mCherry. PCR amplified a characteristic 4.8-kb DNA product from MVA-S genomic DNA compared to vector plasmid DNA (pIII-S). The expected 0.762-kb DNA fragment was obtained from nonrecombinant MVA DNA. (*C*) Multiple-step growth analysis of recombinant MVA-SARS-2-S (MVA-S) and nonrecombinant MVA (MVA). Differences in virus growth were determined by area under curve (AUC) prior to analysis by one-way ANOVA test. Error bars indicate the interquartile range (IQR) from the median. Asterisks represent statistically significant differences between groups: ns, nonsignificant; ****P < 0.0001.

cDNA containing the entire gene sequence encoding SARS-CoV-2-S protein (SARS-2-S) from the virus isolate Wuhan HU-1 (GenBank accession no. MN908947.1) was placed under the transcriptional control of the enhanced synthetic vaccinia virus early/late promoter PmH5 (27) in the MVA vector plasmid pIIIH5red-SARS-2-S, and introduced by homologous recombination into deletion site III in the MVA genome (Fig. 1*A*). Clonal recombinant MVA viruses expressing SARS-2-S (MVA-SARS-2-S) were isolated in repetitive plaque purification using transient coproduction of the fluorescent marker protein mCherry to screen for red fluorescent cell foci (22, 28). PCR analysis of viral DNA confirmed the genetic integrity of the recombinant viruses demonstrating the site-specific insertion of the heterologous SARS-2-S gene sequences in the MVA genome, and subsequently the proper removal of the mCherry marker gene from the genome of final recombinant viruses (Fig. 1*B*). MVA-SARS-2-S virus isolates were genetically stable and showed the expected MVA-specific genetics with regard to characteristic deletions and sequence alterations in the MVA genome (*SI Appendix*, Fig. S1). The recombinant viruses replicated efficiently in the chicken embryo fibroblast cell line DF-1, but not in the human cell lines HeLa, A549 or HaCat (Fig. 1*C*).

### Characterization of SARS-CoV-2 S Protein Expressed by Recombinant MVA.

**Fig. 2.**
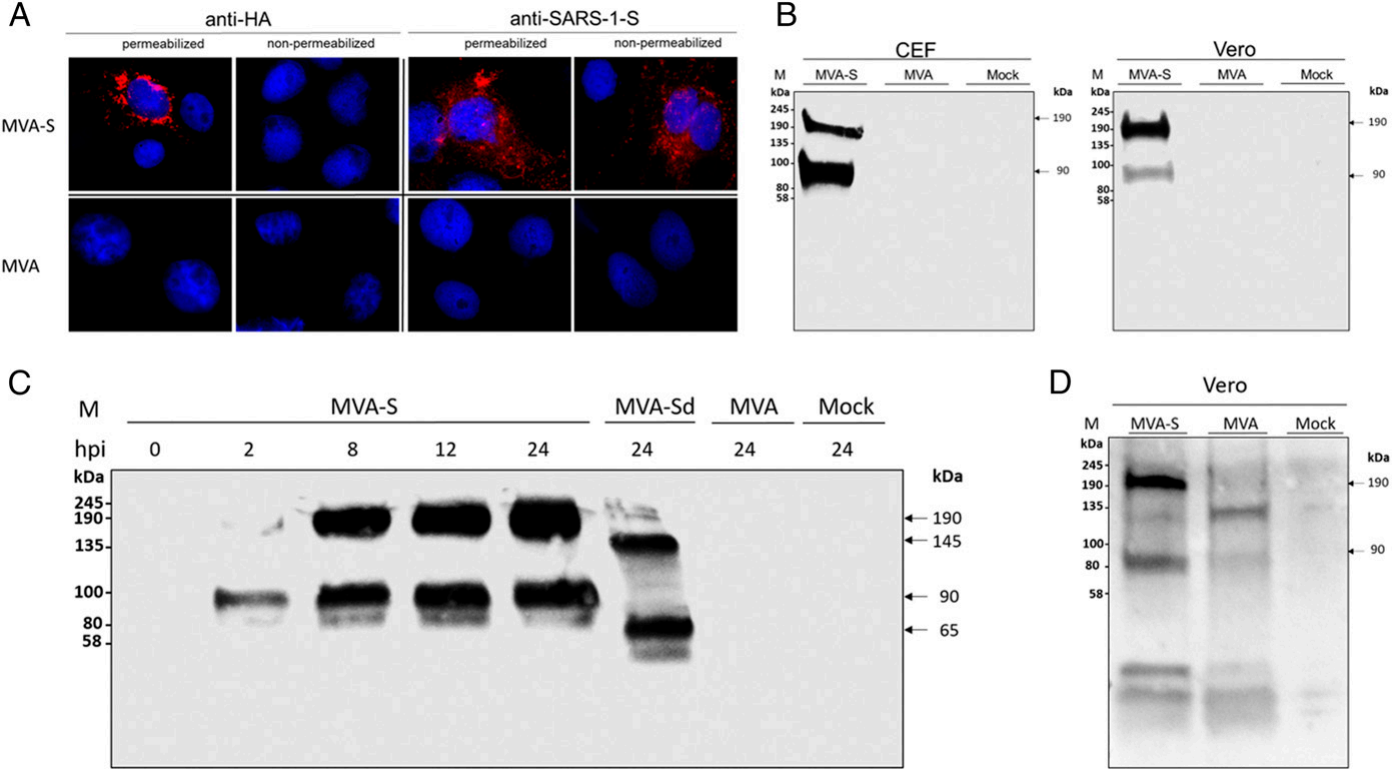
Synthesis of full-length S glycoprotein in MVA-SARS-2-S (MVA-S)–infected cells. (*A*) Permeabilized or nonpermeabilized infected cells were probed with monoclonal antibodies directed against the HA-tag or the S protein of SARS-CoV (SARS-1-S). Polyclonal goat anti-mouse antibody served for S-specific fluorescent staining (red). Cell nuclei were counterstained with DAPI (blue). (*B*) Chicken embryonic fibroblasts (CEFs) and Vero cells were infected with a multiplicity of infection (MOI) of 10 and collected 24 h postinfection (hpi). (*C* and *D*) Vero cells were infected with MVA-SARS-2-S (MVA-S) at a MOI of 10 and collected at indicated time points. PNGase F was used for deglycosylation (MVA-Sd). Polypeptides in cell lysates were separated by SDS-PAGE and analyzed with a monoclonal antibody against the HA-tag (1:8,000) (*B* and *C*) or with human serum (1:200) (*D*). Lysates from noninfected (Mock) or nonrecombinant MVA-infected (MVA) cells were used as controls.

To determine the expression pattern of the recombinant SARS-CoV-2 S protein, we stained MVA-SARS-2-S–infected Vero cells with HA-tag- or S-specific monoclonal antibodies and analyzed them using fluorescence microscopy. A mouse monoclonal antibody directed against the 9-aa HA-tag at the C terminus of the recombinant SARS-2-S protein revealed highly specific staining in permeabilized cells corresponding to the expected intracellular localization of the S-protein C-terminal end. A SARS-CoV-1-S–specific monoclonal antibody showing cross-reactivity with SARS-CoV-2 (29) in recognizing an epitope in the external domain of the SARS-CoV-2-S protein also allowed the specific staining of nonpermeabilized MVA-SARS-2-S–infected Vero cells, suggesting that the SARS-2-S protein was readily translocated to the plasma membrane (Fig. 2*A*).

To examine the MVA-produced recombinant S protein in more detail, we prepared total lysates from MVA-SARS-2-S–infected chicken embryonic fibroblasts (CEFs) or Vero cells for separation by SDS-PAGE and subsequent immunoblot analysis (Fig. 2). The mouse monoclonal antibody directed against the HA-tag at the C terminus of the recombinant SARS-2-S protein revealed two prominent protein bands that migrated with molecular masses of ∼190 and 90–100 kDa (Fig. 2*B*). As in the SDS-PAGE the detected protein bands migrated at molecular masses significantly higher than the 145 kDa predicted for full-length SARS-CoV-2-S protein based on its amino acid sequence, we hypothesized that the proteins might be glycosylated. Indeed, NetNGlyc 1.0 server analysis indicated the presence of at least 17 N-glycosylation sites for co- and posttranslational modifications. The treatment of cell lysates with peptide-N-glycosidase F (PNGase F), which removes all N-linked oligosaccharide chains from glycoproteins, reduced the molecular masses of the recombinant S protein bands from 190 to 145 kDa and from 90 to 100 to 65 kDa, matching the expected sizes of unmodified SARS-CoV-2 S and the S2 cleavage product, respectively (Fig. 2*C*).

Interestingly, the protein band corresponding to the S2 cleavage product was more prominent in the lysates from MVA-SARS-2-S–infected CEF cells, whereas lysates from MVA-SARS-2-S–infected Vero cells contained more full-length protein, suggesting host cell-specific differences in the proteolytic cleavage of the S protein (Fig. 2*B*). Importantly, both isoforms were detectable as early as 2 h postinfection (hpi), indicating proper early transcription from the synthetic MVA promoter PmH5, and their amount increased up to 24 hpi, consistent with the timing of abundant vaccinia viral late protein synthesis (Fig. 2*C*). Moreover, antibodies from a COVID-19 patient hospitalized with pneumonia also revealed protein bands corresponding to the molecular masses of full-length S and the S2 polypeptides (Fig. 2*D*).

### MVA-SARS-2-S Induced Antibody Responses in Mice.

**Fig. 3.**
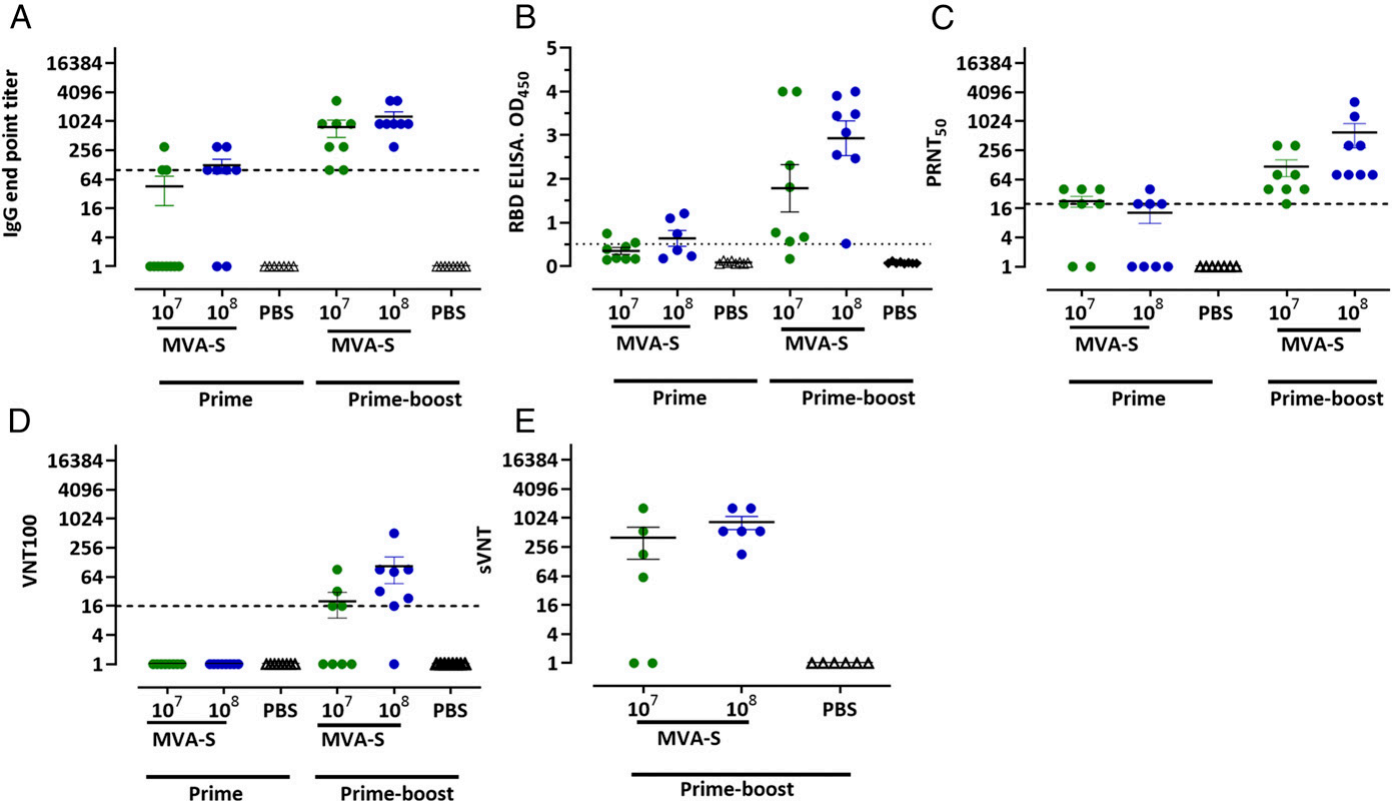
Antigen-specific humoral immunity induced by MVA-SARS-2-S (MVA-S). BALB/c mice were i.m. vaccinated in a prime-boost regime (21-d interval) with 10^7^ or 10^8^ PFU of MVA-S. Mice inoculated with saline (PBS) served as controls. Sera were collected 18 d after the first immunization (prime *n* = 7–8) and 14 d after the second immunization (prime-boost *n* = 6–8). (*A* and *B*) Sera were analyzed for S-specific IgG by ELISA and (*C*–*E*) SARS-CoV-2 neutralizing antibodies by plaque reduction assay (PRNT_50_), virus neutralization (VNT_100_), or surrogate virus neutralization test (sVNT).

To evaluate whether MVA-SARS-2-S induces SARS-CoV-2–specific antibodies, we vaccinated BALB/c mice with a low dose (LD) or high dose (HD) of MVA-SARS-2-S (10^7^ or 10^8^ plaque-forming units [PFU], respectively) using intramuscular (i.m.) administration and prime-boost immunization schedules with a 3-wk interval (Fig. 3 and *SI Appendix*, Fig. S2*A*). At day 18 after the prime inoculation, we detected serum IgG antibodies binding to whole recombinant SARS-CoV-2 S protein in the sera from three of eight LD-vaccinated and four of six HD-vaccinated animals by enzyme-linked immunosorbent assay (ELISA) (Fig. 3*A*). Following the booster immunization on day 21, all vaccinated animals mounted high levels of S-binding serum IgG antibodies with mean titers of 1:900 for the LD vaccination group and 1:1,257 for the HD group (Fig. 3*A*). Importantly, sera from vaccinated mice also contained antibodies binding to the S protein receptor-binding domain (RBD). Already at day 18 post priming, the RBD-binding antibodies were detected in 33% of the mice in the LD dose group (two of six mice; mean OD value, 0.35) and 50% of the mice receiving the HD immunization (three of six; mean OD, 0.63). The boost vaccinations increased the levels of RBD-specific antibodies with 87.5% seropositive mice in the 10^7^ dose group (seven of eight; mean OD, 1.81) and 100% of the animals vaccinated with 10^8^ PFU MVA-SARS-2-S (eight of eight; mean OD, 2.92) (Fig. 3*B*). Since live virus neutralization is the gold standard for coronavirus serologic analysis, we next assessed the mouse sera in two different assays for SARS-CoV-2 neutralization, a plaque reduction neutralization test 50 (PRNT_50_) (30) and a complete virus neutralization test (VNT_100_) (8) (Fig. 3 *C* and *D*). On day 18 following prime immunization, the PRNT_50_ revealed low amounts of SARS-CoV-2 neutralizing antibodies in 50–80% of the sera from vaccinated animals (PRNT_50_ titers of 20–40 for both dose groups). After the boost vaccinations, we detected neutralizing activities in all sera from MVA-SARS-2-S–vaccinated mice with average PRNT_50_ titers of 117 (LD) and 600 (HD) (Fig. 3*C*). Using the VNT_100_ assay, we detected neutralizing activities in 79% of all sera following MVA-SARS-2-S booster immunizations with mean reciprocal titers of 19.8 (four of six seropositive mice, LD group) and 105.8 (seven of eight mice, HD group) (Fig. 3*D*). We obtained similar results when testing the sera in a recently established high-throughput surrogate virus neutralization test for SARS-CoV-2 (sVNT) (31). After the boost immunizations on day 21, we detected levels of surrogate neutralizing antibodies with mean titers of 400 (four of six seropositive mice, LD) and 840 (six of six, HD) (Fig. 3*E* and *SI Appendix*, Fig. S3 *A–C*). Altogether, these results indicate that both LD and HD prime-boost immunization protocols induce a robust anti–SARS-CoV-2-S humoral response and resulted in the generation of neutralizing anti–SARS-CoV-2-S antibodies.

### MVA-SARS-2-S Induced T Cell Responses in Mice.

**Fig. 4.**
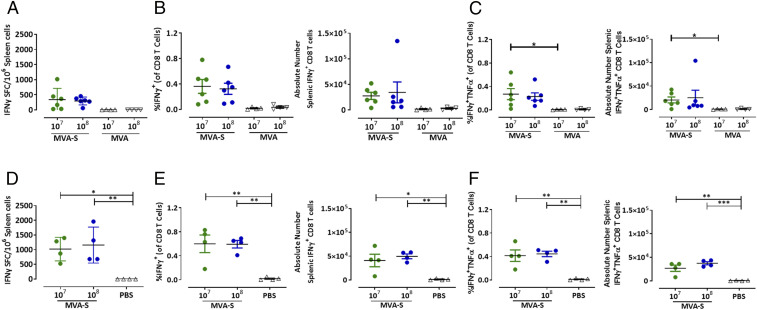
Activation of S-specific CD8^+^ T cells after prime-boost immunization with MVA-SARS-2-S. Groups of BALB/c mice were i.m. immunized twice with 10^7^ or 10^8^ PFU MVA-SARS-2-S (MVA-S). Mock-immunized mice (PBS) were negative controls. (*A*–*C*) Splenocytes (*n* = 6) were collected and prepared on day 8 after prime, or (*D*–*F*) boost immunization on day 21 (*n* = 4). Splenocytes were stimulated with the H2-K^d^–restricted peptide S_268–276_ (S1; GYLQPRTFL) and tested by IFN-γ ELISPOT assay and IFN-γ/TNF-α ICS plus FACS analysis. (*A* and *D*) IFN-γ SFCs measured by ELISPOT assay. (*B* and *E*) IFN-γ–producing CD8^+^ T cells measured by FACS analysis. Graphs show the frequency and absolute number of IFN-γ^+^ CD8^+^ T cells. (*C* and *F*) IFN-γ– and TNF-α–producing CD8^+^ T cells measured by FACS analysis. Graphs show the frequency and absolute number of IFN-γ^+^ TNF-α^+^ CD8^+^ T cells. Differences between groups were analyzed by one-way ANOVA and Tukey post hoc test. Asterisks represent statistically significant differences between two groups: *P < 0.05, **P < 0.01, and ***P < 0.001.

To assess the activation of SARS-CoV-2–specific cellular immunity, we monitored S-specific CD8^+^ and CD4^+^ T cells in BALB/c mice vaccinated with LD or HD MVA-SARS-2-S in prime and prime-boost immunization schedules using 3-wk intervals (*SI Appendix*, Fig. S2*A*). To assess S antigen-specific cellular responses by interferon-γ (IFN-γ) ELISPOT, we isolated splenocytes at day 8 after MVA-SARS-2-S prime or prime-boost immunization and used S-specific peptide stimulation for activation upon in vitro culture. Since information is limited on antigen specificities of SARS-CoV-2–specific T cells, we screened the Immune Epitope Database (IEDB) to select putative S-specific peptide epitopes compatible with activation of CD8^+^ or CD4^+^ T cells (*SI Appendix*, Tables S2 and S3). When testing pools of the predicted peptides with splenocytes from BALB/c mice immunized with 10^8^ PFU of MVA-SARS-2-S, we detected responses above background in several peptide pools and identified the immunodominant SARS-CoV-2 S H2-K^d^ epitope S_269–278_ (GYLQPRTFL; S1 N-terminal domain, *SI Appendix*, Fig. S4). To evaluate the primary activation of SARS-2-S epitope-specific CD8^+^ T cells, we inoculated BALB/c mice once with LD or HD MVA-SARS-2 and analyzed splenocytes on day 8 after vaccination. Single i.m. immunizations with MVA-SARS-2-S already induced S_269–278_-epitope–specific activated CD8^+^ T cells with mean numbers of 341 IFN-γ spot-forming cells (SFCs) in 10^6^ splenocytes for LD and 275 SFCs for HD compared to control mice immunized with nonrecombinant MVA (no SFCs detectable) (Fig. 4*A*). Enzyme-linked immunospot (ELISPOT) data aligned well with fluorescence-activated cell sorting (FACS) analysis of T cells stained for intracellular IFN-γ, where we also found higher frequencies (means of 0.32–0.36%) and higher absolute numbers of IFN-γ^+^ CD8^+^ T cells in splenocytes from vaccinated animals compared to control mice (Fig. 4*B*). Substantial numbers of the activated IFN-γ^+^ CD8^+^ T cells also coexpressed TNF-α (means of 0.22% and 0.27% from total CD8^+^ T cells) (Fig. 4*C*). Of note, mice immunized with LD or HD of MVA-SARS-2-S mounted similar amounts of SARS-2-S–specific CD8^+^ T cells.

The booster immunizations on day 21 further increased the magnitudes of S-specific CD8^+^ T cells in response to MVA-SARS-2-S vaccination. At day 8 post boost, ELISPOT analysis revealed means of 1,020 IFN-γ SFCs in LD-vaccinated mice and 1,159 IFN-γ SFCs in animals receiving HD MVA-SARS-2-S (Fig. 4*D*). Intracellular FACS analysis identified frequencies of 0.62% or 0.60% and absolute numbers of 40,873 or 49,553 IFN-γ^+^ CD8^+^ T cells for mice immunized with LD or HD MVA-SARS-2-S (Fig. 4*E*). Again, we confirmed that the majority (∼75%) of IFN-γ^+^ CD8^+^ T cells also expressed TNF-α (Fig. 4*F*). Spectral flow cytometry of T cell subsets revealed high levels of CD8^+^ effector memory T cells, reduced numbers of naive CD4^+^ T cells, and balanced populations of T helper cells in splenocytes from MVA-SARS-2-S or nonrecombinant MVA-immunized animals (*SI Appendix*, Fig. S8 *A–E*). The MVA-specific immunodominant CD8^+^ T cell determinant F2_26–34_ [SPGAAGYDL (32)] served as a control peptide for the detection and comparative analysis of MVA vector-specific CD8^+^ T cells in BALB/c mice (*SI Appendix*, Figs. S5 *A–C* and S6 *A–C*). In addition, we used S-protein–derived peptides with predicted capacity for MHC II binding to monitor for the presence of activated CD4^+^ T cells. Using three different peptide pools (*SI Appendix*, Table S3), we confirmed the presence of spike-specific CD4^+^ T cells in the spleens of mice immunized with LD and HD prime-boost regimens (*SI Appendix*, Fig. S9).

### Protective Capacity of MVA-SARS-2-S upon SARS-CoV-2 Challenge.

**Fig. 5.**
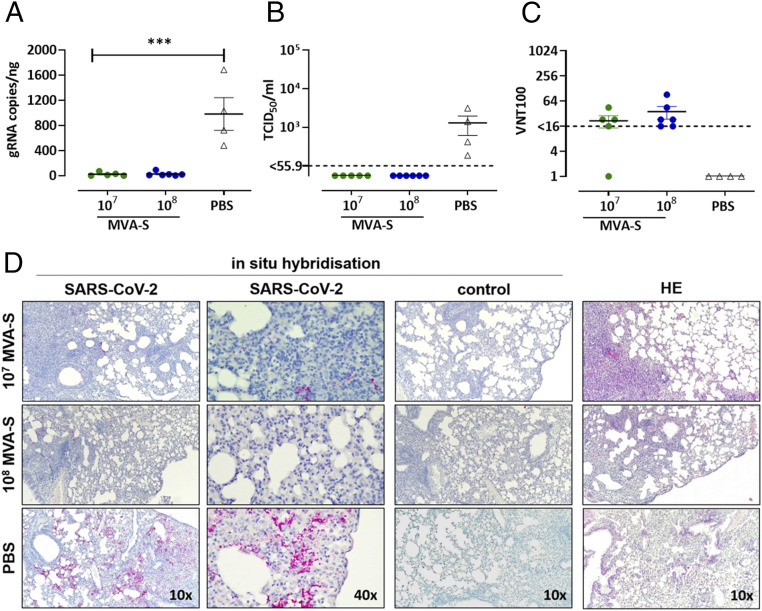
Protective capacity of MVA-SARS-2-S immunization against SARS-CoV-2 infection in human ACE2-transduced (hACE2) BALB/c mice. Groups of BALB/c mice (*n* = 4–6) were i.m. immunized twice with 10^7^ or 10^8^ PFU of MVA-SARS-2-S (MVA-S) over a 21-d interval. Mock-immunized mice (PBS) served as controls. About 2 wk after the last immunization, mice were sensitized with an adenovirus expressing hACE2 and mCherry and infected with SARS-CoV-2 3 d after transduction. Four days post challenge, the animals were killed and samples were taken for further analysis. (*A*) Lung tissues were harvested to determine SARS-CoV-2 gRNA copies, (*B*) the amounts of infectious SARS-CoV-2 by TCID_50_/mL, and (*D*) lung histopathology. (*C*) Sera were tested for SARS-CoV-2 neutralizing antibodies by virus neutralization (VNT_100_). (*D*) Fixed tissue was stained with hematoxylin and eosin (HE) or with in situ probes. Images show medium- (10×) and high-power (40×) magnification; images are representative of *n* = 4–6 per group. Statistical evaluation was performed with GraphPad Prism for Windows. Statistical significance of differences between groups is indicated as follows: ***P < 0.001.

To model productive infection with SARS-CoV-2, we used an adenoviral transduction-based mouse model similar to those described recently (33, 34). We intratracheally transduced MVA-SARS-2-S–vaccinated BALB/c mice with 5 × 10^8^ PFU of an adenoviral vector encoding both the human ACE2 receptor and the marker protein mCherry (ViraQuest) at about 2 wk after prime-boost immunization. Three days later, the animals were infected with 1.5 × 10^4^ tissue culture infectious dose 50 (TCID_50_) SARS-CoV-2 (isolate BavPat1/2020 isolate, European Virus Archive Global #026V-03883). Daily, body weight, spontaneous behavior, and general condition of the mice were monitored and summarized in a clinical score. No clinical abnormalities were observed (*SI Appendix*, Fig. S10 *A* and *B*). Four days post challenge, the animals were killed, blood samples taken, and the lungs harvested to measure viral loads. Substantial virus RNA loads were found in mock-immunized control mice. In contrast, the lung tissue of both LD and HD MVA-SARS-2-S–immunized animals contained significantly lower levels of SARS-CoV-2 RNA (<100 genome equivalents/ng total RNA; Fig. 5*A*). Adenoviral vector transduction levels of lung tissues were analyzed by real-time RT-PCR analysis to confirm comparable amounts of mCherry RNA (*SI Appendix*, Fig. S10*C*). In addition, we detected >1,000 TCID_50_/mL infectious SARS-CoV-2 in the lungs from control mice but not in the lungs of immunized mice, indicating the efficient inhibition of SARS-CoV-2 replication by vaccine-induced immune responses (Fig. 5*B*). In agreement with these data, only sera from MVA-SARS-2-S–vaccinated animals (10 of 11) contained SARS-CoV-2 neutralizing circulating antibodies (Fig. 5*C*). Lung histopathology was evaluated after hematoxylin–eosin (HE) staining. In lungs of PBS mock-vaccinated control mice, we observed interstitial pneumonia displaying multiple, partially confluent foci with mainly lymphohistiocytic infiltrations in the alveolar interstitium with emphasis on the peribronchiolar and perivascular regions. Nevertheless, no drastic reduction of lung lesions in terms of severity and extension was observed in the vaccinated groups. Lungs of vaccinated mice showed mild hyperplasia of the bronchus-associated lymphatic tissue and mild cuffs of lymphocytes around blood vessels. However, in situ hybridization revealed a clear reduction of SARS-CoV-2 RNA in the lungs of both vaccinated groups, characterized by reduced or absent red staining for SARS-CoV-2 RNA, consistent with RT-PCR results. In contrast, lung slides of PBS mock-vaccinated animals showed extensive red staining, especially in severely affected areas (Fig. 5*D*).

## Discussion

Here, we report that the COVID-19 candidate vaccine MVA-SARS-2-S is compatible with clinical use and industrial-scale production. Building on extensive prior experience developing a candidate vaccine against MERS (22–24, 26), we selected the full-length SARS-CoV-2 S protein for delivery by recombinant MVA. The vector virus replicated efficiently in DF-1 cells, the cell substrate for an optimized manufacturing process, and MVA-SARS-2-S stably produces S-protein antigen upon serial amplifications at low multiplicities of infection.

Similar to our experience gained with MVA-MERS-S, expression of the SARS-CoV-2 S gene by recombinant MVA resulted in a glycoprotein with a molecular mass of about 190 kDa. Treatment with glycosidase to remove all N-linked carbohydrates produced a polypeptide of 145 kDa, closely corresponding to the molecular weight predicted from S gene nucleotide sequence. In addition, we observed proteolytic cleavage of the full-length SARS-CoV-2 S polypeptide into S1 and S2, apparently with various efficiencies of proteolytic processing depending on the cell substrate used. This finding is in agreement with previous reports suggesting complex activation of the betacoronavirus S proteins, including the involvement of multiple cleavage events and several host proteases (35, 36). Similar to our findings with the MVA-encoded MERS-CoV S protein, SARS-CoV-2-S–specific detection by immunofluorescence included strong surface staining of MVA-SARS-2-S–infected cells. We conclude that the recombinant SARS-CoV-2 S protein is transported through the Golgi apparatus and is expressed at the cell surface, as shown previously for functional S produced from plasmid expression vectors (7, 37, 38).

Since the biochemical characterization of the MVA-expressed S suggested production of a mature and properly folded spike antigen, we investigated whether MVA-SARS-2-S elicits S-specific immune responses. In proof-of-principle experiments, mice receiving the MVA-SARS-2-S vaccine twice intramuscularly developed circulating S-specific antibodies that neutralized SARS-CoV-2 infections in cell culture and mounted high levels of SARS-CoV-2-S–specific CD8^+^ T cells. MVA-SARS-2-S elicited levels of virus neutralizing antibodies in BALB/c mice that were comparable to those induced by ChAdOx1 nCoV-19 or MVA-MERS-S vaccinations (23, 39), and evidence from preclinical studies in nonhuman primates and hamsters indicates that vaccine-induced SARS-CoV-2 neutralizing antibodies correlate with protection against lung infection and clinical disease (40–42). The humoral immune responses elicited by MVA-SARS-2-S was measured by ELISA, two different SARS-CoV-2 neutralization assays, and a surrogate neutralization assay; all results pointed to a clear benefit of the booster immunization. These data are in line with results from phase 1 clinical testing of our MVA-MERS-S candidate vaccine providing evidence of humoral immunogenicity using homologous prime-boost vaccination (26). For SARS-CoV-2 neutralizing antibodies, we found a strong correlation between the results obtained from authentic virus neutralization (PRNT_50_, VNT_100_) and the data from surrogate neutralization (sVNT) using a high-throughput and BSL-2/3-sparing test. These data corroborate the findings of a recent study comparing this high-throughput sVNT assay to a pseudotyped virus neutralization assay based on SARS-CoV-2 S protein-carrying vesicular stomatitis virus (31).

The i.m. immunizations of BALB/c mice with low- and high-dose MVA-SARS-2-S induced robust and nearly equal amounts of SARS-S–specific CD8^+^ T cells in prime and prime-boost vaccination. The average number of S-specific T cells was comparable to the average number of MVA vector-specific T cells highlighting the strong immunogenicity of MVA-SARS-2-S for inducing an S-specific CD8^+^ T cell response. Recent data demonstrated that activation of a strong TH1 cell response has been associated with less severe cases of COVID-19, whereas TH2 cell responses have been associated with more severe lung disease in humans (43). Thus, COVID-19 vaccine candidates should preferably activate a TH1 cell-like phenotype. Our prime-boost vaccinations with MVA-SARS-2-S did not impair the balanced T helper cell populations monitored in BALB/c mice by spectral flow cytometry. Of note, two additional studies also demonstrated the induction of predominantly TH1-type immune responses in mice immunized with MVA vector vaccines encoding SARS-CoV-2 S antigens (44, 45). The importance of vaccine-induced T cell responses is illustrated by studies not only monitoring the adaptive immunity to SARS-CoV-2 in patients, but also demonstrating that strong SARS-CoV-2–specific CD4^+^ or CD8^+^ T cell responses are associated with low disease severity in individuals with COVID-19 (5). Of note, MVA-SARS-2-S vaccination activated a high number of CD8^+^ T cells secreting both IFN-γ and TNF-α. Previously, in the context of HIV infection, this subset of bifunctional CD8^+^ T cells has been shown to be more strongly associated with cytotoxic activity compared to CD8^+^ T cells secreting solely IFN-γ (46). Recent studies also demonstrated an abundance of polyfunctional CD8^+^ T cells in SARS-CoV-2–infected individuals with asymptomatic or mild COVID-19 disease (47).

In a mouse model of SARS-CoV-2 lung infection, all vaccinated BALB/c mice exhibited little or no replication of SARS-CoV-2, irrespective of whether low- or high-dose MVA-SARS-2-S was used for vaccination. Particularly encouraging was the complete absence of detectable infectious virus in the lungs of immunized animals. Notably, we found no evidence of a potential enhancement of SARS-CoV-2 infection through S-antigen–specific antibody induction, confirming our data with MVA-MERS-S that the S glycoprotein is an important and safe vaccine antigen (23, 24). These results together with the data from disease and pathology monitoring upon immunization (*SI Appendix*, Fig. S2 *B* and *C*) provided valuable evidence of MVA-SARS-2-S preclinical safety.

Overall, the MVA-SARS-2-S vector vaccine merits further development and the results presented here provided information for the start of a phase 1 clinical trial on September 30, 2020. To counteract the SARS-CoV-2 pandemic, candidate vaccines are being rapidly investigated in unprecedented numbers, and first front-runner vaccines obtained emergency licensing in Europe and the United States in 2020 (48). However, there is still much to learn when moving forward in COVID-19 vaccination. We expect that optimized protective immunity to COVD-19 will require vaccine approaches eliciting antiviral SARS-CoV-2–specific CD4^+^ and CD8^+^ T cells in a coordinated manner, together with virus neutralizing antibodies, in various population groups including children, the elderly, and individuals with comorbidities.

## Materials and Methods

Detailed procedures and sources of reagents are described in *SI Appendix*.

### Generation of Recombinant Viruses.

The coding sequence of the full-length SARS-CoV-2 S protein was modified *in silico* by introducing silent mutations to remove runs of guanines or cytosines and termination signals of vaccinia virus-specific early transcription, and to add a C-terminal HA-tag sequence encoding nine amino acids (YPYDVPDYA, amino acids 98–106 from influenza virus). The cDNA was produced by DNA synthesis and cloned into the MVA transfer plasmid pIIIH5red under transcriptional control of the synthetic vaccinia virus early/late promoter PmH5. MVA vector viruses were obtained following the established protocols for vaccine development as described in previous studies (22). MVA (clonal isolate MVA-F6-sfMR) was grown on CEFs under serum-free conditions and served as a nonrecombinant backbone virus to construct MVA vector viruses expressing the SARS-CoV-2 S gene sequences. To obtain vaccine preparations, recombinant MVA-SARS-2-S were amplified on CEF or DF-1 cell monolayers, purified by ultracentrifugation through sucrose and reconstituted to high-titer stock preparations. PFUs were counted to determine viral titers (28).

For use of patient serum, approval for the full study protocol was obtained from the Ethics Committee at the Medical Faculty of Ludwig Maximilian University of Munich (LMU Munich) (vote 20-225 KB) in accordance with the guidelines of the Declaration of Helsinki. All patients gave written informed consent and data have been used in an anonymized form.

### Vaccination Experiments in Mice.

BALB/c mice were purchased from Charles River Laboratories and maintained under specified pathogen-free conditions. All animal experiments were handled in compliance with the European and national regulations for animal experimentation (European Directive 2010/63/EU; Animal Welfare Acts in Germany). Immunizations were performed using intramuscular applications with 10^7^ or 10^8^ PFU recombinant MVA-SARS-2-S, nonrecombinant MVA, or PBS (mock) into the quadriceps muscle of the left hind leg. Blood was collected on days 0, 18, or 35. Coagulated blood was centrifuged at 1,300 × *g* for 5 min to separate serum.

### Transduction of Vaccinated Mice with Ad_ACE2-mCherry and Challenge Infection with SARS-CoV-2.

Vaccinated mice underwent intratracheal inoculation with 5 × 10^8^ PFU Adenovirus-ACE2-mCherry under ketamine/xylazine anesthesia. Three days post transduction, mice were infected via the intranasal route with 1.5 × 10^4^ tissue culture infectious dose 50 (TCID_50_) SARS-CoV-2 (BavPat1/2020 isolate, European Virus Archive Global #026V-03883). Mice were killed 4 d postinfection, and serum as well as lung tissue samples were analyzed for virus loads.

### Quantitative Real-Time RT-PCR to Determine SARS-CoV-2 or mCherry RNA.

Tissue samples of immunized and challenged mice were excised from the left lung lobes and homogenized in 1 mL of Dulbecco’s modified Eagle’s medium. SARS-CoV-2 titers in supernatants (in TCID_50_ per milliliter) were determined on VeroE6 cells. RNA isolation was performed with the RNeasy minikit. The RNA amount was measured using the NanoDrop ND-100 spectrophotometer. Total RNA was reverse transcribed and quantified by real-time PCR using the OneStep RT-PCR kit. Additionally, for every tissue sample from transduced and infected mice, evidence for successful ACE2 transduction was determined by real-time RT-PCR for mCherry mRNA with the OneStep RT-PCR kit. Quantification was carried out using a standard curve based on 10-fold serial dilutions of appropriate control RNA ranging from 10^2^ to 10^5^ copies.

### Histopathological Examination of Lung Tissue.

Lungs were collected on day 4 post challenge with SARS-CoV-2 and processed for histological analysis. Briefly, tissue was fixed in formalin and embedded in paraffin. Four-micrometer sections were cut with a microtome and stained with HE. To investigate the presence of viral RNA in lung tissue by in situ hybridization, a RNA-specific probe, targeted against the S gene of the SARS-CoV-2, was hybridized. Afterward, signal amplification was performed and alkaline-phosphatase–labeled probes were used in combination with Fast Red substrate allowing signal detection.

### Antigen-Specific IgG ELISA.

For analysis of SARS-2-S–specific serum IgG titers, flat-bottom 96-well ELISA plates were coated with 50 ng/well recombinant S protein. Mouse sera were serially diluted in PBS/BSA. Plates were then incubated, washed, and probed with goat anti-mouse IgG HRP diluted in PBS/BSA, and developed with 3,3′,5,5′-tetramethylbenzidine (TMB) as chromogenic substrate. The absorbance of each serum sample was measured at 450 nm with a 620-nm reference wavelength. ELISA data were normalized using the positive control. The cutoff value for positive mouse serum samples was determined by calculating the mean of the normalized OD 450-nm values of the PBS control group sera plus 6 SDs (mean + 6SD).

### RBD-Specific IgG ELISA.

RBD-specific serum IgG titres were measured as previously described (30). After blocking, serum dilutions (diluted 1:100) were incubated at 37 °C for 1 h. Antigen-specific antibodies were detected by using peroxidase-labeled rabbit anti-mouse IgG and TMB as a substrate. Absorbance was measured at 450 nm. A cutoff was set at an OD of 0.5.

### sVNT.

To test for the presence of neutralizing anti-SARS-CoV-2-S serum antibodies, we used surrogate virus neutralization test as described before with slight modifications (31). Briefly, SARS-CoV-2 S RBD was preincubated with heat-inactivated test sera. Afterward, SARS-CoV-2 S RBD-serum mixtures were loaded onto 96-well plates coated with ACE2 [produced in-house as described by Bošnjak et al. (31)], blocked, and incubated for 1 h at 37 °C. After washing plates were incubated with HRP-conjugated anti–His-tag antibody and developed by addition of TMB. The optical density values measured at 450 and 570 nm were used to calculate percentage of inhibition after subtraction of background values as inhibition (%) = (1 − Sample OD value/Average SARS-CoV-2 S RBD OD value) × 100. To remove background effects, the mean percentage of inhibition from nonspecific mouse serum (Invitrogen) was deducted from sample values and neutralizing anti-SARS-CoV2-S antibodies titers were determined as serum dilution that still had binding reduction > mean + 2 SD of values from sera of vehicle-treated mice.

### PRNT_50_.

Neutralization capacity against SARS-CoV-2 (German isolate; GISAID ID EPI_ISL 406862; European Virus Archive Global #026V-03883) was tested as described previously (30). We twofold serially diluted heat-inactivated serum samples in Dulbecco’s modified Eagle’s medium starting at a dilution of 1:10 in 50 μL. We then added 50 μL of virus suspension (400 PFU) to each well and incubated at 37 °C for 1 h before placing the mixtures on VeroE6 cells. After incubation for 1 h, we washed cells supplemented with medium, and incubated them for 8 h. After incubation, we fixed the cells with 4% formaldehyde/PBS and stained the cells with polyclonal rabbit anti-SARS-CoV antibody and a secondary peroxidase-labeled goat anti-rabbit IgG. We developed the signal using a precipitate forming TMB substrate and counted the number of infected cells per well by using an ImmunoSpot Image Analyzer. The serum neutralization titer is the reciprocal of the highest dilution resulting in an infection reduction of >50% (PRNT_50_). We considered a titer >20 to be positive.

### SARS-CoV-2 VNT_100_.

The neutralizing activity of mouse serum antibodies was investigated based on a previously published protocol (8). Samples were serially diluted in 96-well plates starting from a 1:16 serum dilution. Samples were incubated for 1 h at 37 °C together with 100 50% tissue culture infectious doses (TCID_50_) of SARS-CoV-2 (BavPat1/2020 isolate, European Virus Archive Global #026V-03883). Cytopathic effects (CPEs) on VeroE6 cells were analyzed 4 d after infection. Neutralization was defined as the absence of CPEs compared to virus controls. For each test, a positive control (neutralizing COVID-19 patient plasma) was used in duplicates as an interassay neutralization standard. Ethical approval was granted by the Ethics Committee at the Medical Faculty of LMU Munich (vote 20-225 KB) in accordance with the guidelines of the Declaration of Helsinki.

### Prediction and Generation of Synthetic SARS-2-S Peptides.

The SARS-CoV-2 S protein (National Center for Biotechnology Information ID: QHD43416.1; Uniprot ID: P0DTC2 [SPIKE_SARS2]) served for epitope prediction, and probable CD8^+^ and CD4^+^ T cell determinants were examined with the Immune Epitope Database and Analysis Resource (IEDB) (https://www.iedb.org/). For identification of potential CD8^+^ T cell determinants, the MHC-I Binding Prediction and MHC-I Processing Prediction tools (49) were used. To confirm that peptides were potential binders of MHC class I alleles H2-K^d^, H2-D^d^, and H2-L^d^, they were further screened for MHC I binding using the RankPep server (50). Peptides that were found to bind to any of the above alleles were selected for synthesis and testing. All peptides were dissolved in PBS or DMSO, aliquoted, and stored at -20 °C.

### Analysis of Cellular Response by ELISPOT.

At days 8 and 14 post prime or prime-boost vaccination, splenocytes were prepared and enzyme-linked immunospot (ELISPOT) assay served to measure IFN-γ–producing cells. Splenocytes were seeded in 96-well plates and stimulated with peptides. Nonstimulated cells and cells stimulated with phorbol myristate acetate/ionomycin or vaccinia virus peptide SPGAAGYD [F2_26–34_ (32)] served as controls. After incubation, plates were stained and spots were counted and analyzed by using an automated ELISPOT plate reader.

### T Cell Analysis by Intracellular Cytokine Staining.

For intracellular cytokine staining (ICS), cells were stimulated with S_269–278_ peptide or vaccinia virus peptide F2_26–34_ for 2 h. Then, brefeldin A was added and cells were further stimulated for 4 h. After stimulation, cells were stained with anti–CD3-PE, anti-CD4 Brilliant Violet 421, anti-CD8α Alexa Fluor 488, and purified CD16/CD32. Cells were then washed, fixed, permeabilized with Perm Wash buffer, and stained intracellularly anti–IFN-γ plus anti–TNF-α. Data were acquired by a MACSQuant flow cytometer and analyzed using FlowJo software.

## Supplementary Material

Supplementary File

## Data Availability

All study data are included in the article and *SI Appendix*.

## References

[1] Coronaviridae Study Group of the International Committee on Taxonomy of Viruses, The species severe acute respiratory syndrome-related coronavirus: Classifying 2019-nCoV and naming it SARS-CoV-2. Nat. Microbiol. 5, 536–544 (2020). 32123347 10.1038/s41564-020-0695-z PMC7095448

[2] G. A. Poland, I. G. Ovsyannikova, R. B. Kennedy, SARS-CoV-2 immunity: Review and applications to phase 3 vaccine candidates. Lancet 396, 1595–1606 (2020). 33065034 10.1016/S0140-6736(20)32137-1 PMC7553736

[3] S. H. Hodgson ., What defines an efficacious COVID-19 vaccine? A review of the challenges assessing the clinical efficacy of vaccines against SARS-CoV-2. Lancet Infect. Dis. 21, e26–e35 (2020). 33125914 10.1016/S1473-3099(20)30773-8 PMC7837315

[4] M. N. Ramasamy .; Oxford COVID Vaccine Trial Group, Safety and immunogenicity of ChAdOx1 nCoV-19 vaccine administered in a prime-boost regimen in young and old adults (COV002): A single-blind, randomised, controlled, phase 2/3 trial. Lancet 396, 1979–1993 (2021). 33220855 10.1016/S0140-6736(20)32466-1 PMC7674972

[5] C. Rydyznski Moderbacher ., Antigen-specific adaptive immunity to SARS-CoV-2 in acute COVID-19 and associations with age and disease severity. Cell 183, 996–1012.e19 (2020). 33010815 10.1016/j.cell.2020.09.038 PMC7494270

[6] D. Wrapp ., Cryo-EM structure of the 2019-nCoV spike in the prefusion conformation. Science 367, 1260–1263 (2020). 32075877 10.1126/science.abb2507 PMC7164637

[7] M. Hoffmann ., SARS-CoV-2 cell entry depends on ACE2 and TMPRSS2 and is blocked by a clinically proven protease inhibitor. Cell 181, 271–280.e8 (2020). 32142651 10.1016/j.cell.2020.02.052 PMC7102627

[8] C. Kreer ., Longitudinal isolation of potent near-germline SARS-CoV-2-neutralizing antibodies from COVID-19 patients. Cell 182, 843–854.e12 (2020). 32673567 10.1016/j.cell.2020.06.044 PMC7355337

[9] S. J. Zost ., Potently neutralizing and protective human antibodies against SARS-CoV-2. Nature 584, 443–449 (2020). 32668443 10.1038/s41586-020-2548-6 PMC7584396

[10] P. M. Folegatti .; Oxford COVID Vaccine Trial Group, Safety and immunogenicity of the ChAdOx1 nCoV-19 vaccine against SARS-CoV-2: A preliminary report of a phase 1/2, single-blind, randomised controlled trial. Lancet 396, 467–478 (2020). 32702298 10.1016/S0140-6736(20)31604-4 PMC7445431

[11] L. R. Baden .; COVE Study Group, Efficacy and safety of the mRNA-1273 SARS-CoV-2 vaccine. N. Engl. J. Med. 384, 403–416 (2021). 33378609 10.1056/NEJMoa2035389 PMC7787219

[12] M. J. Mulligan ., Phase I/II study of COVID-19 RNA vaccine BNT162b1 in adults. Nature 586, 589–593 (2020). 32785213 10.1038/s41586-020-2639-4

[13] E. E. Walsh ., Safety and immunogenicity of two RNA-based Covid-19 vaccine candidates. N. Engl. J. Med. 383, 2439–2450 (2020). 33053279 10.1056/NEJMoa2027906 PMC7583697

[14] L. A. Jackson .; mRNA-1273 Study Group, An mRNA vaccine against SARS-CoV-2—preliminary report. N. Engl. J. Med. 383, 1920–1931 (2020). 32663912 10.1056/NEJMoa2022483 PMC7377258

[15] US Food and Drug Administration, Moderna COVID-19 vaccine (2021). https://www.fda.gov/emergency-preparedness-and-response/coronavirus-disease-2019-covid-19/moderna-covid-19-vaccine. Accessed 12 April 2021.

[16] European Medicines Agency, EMA recommends first COVID-19 vaccine for authorisation in the EU (21 December 2020). https://www.ema.europa.eu/en/news/ema-recommends-first-covid-19-vaccine-authorisation-eu. Accessed 12 April 2021.

[17] European Commission, Union Register of medicinal products for human use: COVID-19 vaccine Janssen (2021). https://ec.europa.eu/health/documents/community-register/html/h1525.htm. Accessed 12 April 2021.

[18] European Commission, Union Register of medicinal products for human use: Vaxzevria (2021). https://ec.europa.eu/health/documents/community-register/html/h1529.htm. Accessed 12 April 2021.

[19] G. Sutter, B. Moss, Nonreplicating vaccinia vector efficiently expresses recombinant genes. Proc. Natl. Acad. Sci. U.S.A. 89, 10847–10851 (1992). 1438287 10.1073/pnas.89.22.10847 PMC50439

[20] A. Volz, G. Sutter, Modified vaccinia virus Ankara: History, value in basic research, and current perspectives for vaccine development. Adv. Virus Res. 97, 187–243 (2017). 28057259 10.1016/bs.aivir.2016.07.001 PMC7112317

[21] F. Chiuppesi ., Development of a multi-antigenic SARS-CoV-2 vaccine candidate using a synthetic poxvirus platform. Nat. Commun. 11, 6121 (2020). 33257686 10.1038/s41467-020-19819-1 PMC7705736

[22] F. Song ., Middle East respiratory syndrome coronavirus spike protein delivered by modified vaccinia virus Ankara efficiently induces virus-neutralizing antibodies. J. Virol. 87, 11950–11954 (2013). 23986586 10.1128/JVI.01672-13 PMC3807317

[23] A. Volz ., Protective efficacy of recombinant modified vaccinia virus Ankara delivering Middle East respiratory syndrome coronavirus spike glycoprotein. J. Virol. 89, 8651–8656 (2015). 26018172 10.1128/JVI.00614-15 PMC4524222

[24] B. L. Haagmans ., An orthopoxvirus-based vaccine reduces virus excretion after MERS-CoV infection in dromedary camels. Science 351, 77–81 (2016). 26678878 10.1126/science.aad1283

[25] S. Veit, S. Jany, R. Fux, G. Sutter, A. Volz, CD8^+^ T cells responding to the Middle East respiratory syndrome coronavirus nucleocapsid protein delivered by vaccinia virus MVA in mice. Viruses 10, 718 (2018). 10.3390/v10120718 PMC6316859 30558354

[26] T. Koch ., Safety and immunogenicity of a modified vaccinia virus Ankara vector vaccine candidate for Middle East respiratory syndrome: An open-label, phase 1 trial. Lancet Infect. Dis. 20, 827–838 (2020). 32325037 10.1016/S1473-3099(20)30248-6 PMC7172913

[27] L. S. Wyatt, S. T. Shors, B. R. Murphy, B. Moss, Development of a replication-deficient recombinant vaccinia virus vaccine effective against parainfluenza virus 3 infection in an animal model. Vaccine 14, 1451–1458 (1996). 8994321 10.1016/s0264-410x(96)00072-2

[28] M. Kremer ., Easy and efficient protocols for working with recombinant vaccinia virus MVA. Methods Mol. Biol. 890, 59–92 (2012). 22688761 10.1007/978-1-61779-876-4_4

[29] E. Welsh, J. A. Cardenas-de la Garza, A. Cuellar-Barboza, R. Franco-Marquez, R. I. Arvizu-Rivera, SARS-CoV-2 spike protein positivity in pityriasis rosea-like and urticaria-like rashes of COVID-19. Br J. Dermatol 184, 1194–1195 (2021). 33511657 10.1111/bjd.19833 PMC8013476

[30] N. M. A. Okba ., Severe acute respiratory syndrome coronavirus 2-specific antibody responses in coronavirus disease patients. Emerg. Infect. Dis. 26, 1478–1488 (2020). 32267220 10.3201/eid2607.200841 PMC7323511

[31] B. Bošnjak ., Low serum neutralizing anti-SARS-CoV-2 S antibody levels in mildly affected COVID-19 convalescent patients revealed by two different detection methods. Cell. Mol. Immunol. 18, 936–944 (2021). 33139905 10.1038/s41423-020-00573-9 PMC7604543

[32] D. C. Tscharke ., Poxvirus CD8^+^ T-cell determinants and cross-reactivity in BALB/c mice. J. Virol. 80, 6318–6323 (2006). 16775319 10.1128/JVI.00427-06 PMC1488955

[33] J. Sun ., Generation of a broadly useful model for COVID-19 pathogenesis, vaccination, and treatment. Cell 182, 734–743.e5 (2020). 32643603 10.1016/j.cell.2020.06.010 PMC7284240

[34] L.-Y. R. Wong ., Sensitization of non-permissive laboratory mice to SARS-CoV-2 with a replication-deficient adenovirus expressing human ACE2. STAR Protocols 1, 100169 (2020). 33377063 10.1016/j.xpro.2020.100169 PMC7757354

[35] J. A. Jaimes, J. K. Millet, G. R. Whittaker, Proteolytic cleavage of the SARS-CoV-2 spike protein and the role of the novel S1/S2 site. iScience 23, 101212 (2020). 32512386 10.1016/j.isci.2020.101212 PMC7255728

[36] R. J. G. Hulswit, C. A. M. de Haan, B. J. Bosch, Coronavirus spike protein and tropism changes. Adv. Virus Res. 96, 29–57 (2016). 27712627 10.1016/bs.aivir.2016.08.004 PMC7112277

[37] L. Grzelak ., A comparison of four serological assays for detecting anti-SARS-CoV-2 antibodies in human serum samples from different populations. Sci. Transl. Med. 12, eabc3103 (2020). 32817357 10.1126/scitranslmed.abc3103 PMC7665313

[38] J. Buchrieser ., Syncytia formation by SARS-CoV-2-infected cells. EMBO J. 39, e106267 (2020). 33051876 10.15252/embj.2020106267 PMC7646020

[39] N. van Doremalen ., ChAdOx1 nCoV-19 vaccine prevents SARS-CoV-2 pneumonia in rhesus macaques. Nature 586, 578–582 (2020). 32731258 10.1038/s41586-020-2608-y PMC8436420

[40] N. B. Mercado ., Single-shot Ad26 vaccine protects against SARS-CoV-2 in rhesus macaques. Nature 586, 583–588 (2020). 32731257 10.1038/s41586-020-2607-z PMC7581548

[41] K. S. Corbett ., Evaluation of the mRNA-1273 vaccine against SARS-CoV-2 in nonhuman primates. N. Engl. J. Med. 383, 1544–1555 (2020). 32722908 10.1056/NEJMoa2024671 PMC7449230

[42] L. H. Tostanoski ., Ad26 vaccine protects against SARS-CoV-2 severe clinical disease in hamsters. Nat. Med. 26, 1694–1700 (2020). 32884153 10.1038/s41591-020-1070-6 PMC7671939

[43] G. Chen ., Clinical and immunological features of severe and moderate coronavirus disease 2019. J. Clin. Invest. 130, 2620–2629 (2020). 32217835 10.1172/JCI137244 PMC7190990

[44] J. García-Arriaza ., COVID-19 vaccine candidates based on modified vaccinia virus Ankara expressing the SARS-CoV-2 spike induce robust T- and B-cell immune responses and full efficacy in mice. J. Virol. 95, e02260-20 (2021). 10.1128/JVI.02260-20 PMC8092708 33414159

[45] R. Liu ., One or two injections of MVA-vectored vaccine shields hACE2 transgenic mice from SARS-CoV-2 upper and lower respiratory tract infection. Proc. Natl. Acad. Sci. U.S.A. 118, e2026785118 (2021). 33688035 10.1073/pnas.2026785118 PMC8000198

[46] M. Lichterfeld ., HIV-1-specific cytotoxicity is preferentially mediated by a subset of CD8^+^ T cells producing both interferon-γ and tumor necrosis factor-α. Blood 104, 487–494 (2004). 15059848 10.1182/blood-2003-12-4341

[47] T. Sekine .; Karolinska COVID-19 Study Group, Robust T cell immunity in convalescent individuals with asymptomatic or mild COVID-19. Cell 183, 158–168.e14 (2020). 32979941 10.1016/j.cell.2020.08.017 PMC7427556

[48] F. Krammer, SARS-CoV-2 vaccines in development. Nature 586, 516–527 (2020). 32967006 10.1038/s41586-020-2798-3

[49] W. Fleri ., The immune epitope database and analysis resource in epitope discovery and synthetic vaccine design. Front. Immunol. 8, 278 (2017). 28352270 10.3389/fimmu.2017.00278 PMC5348633

[50] P. A. Reche, J.-P. Glutting, H. Zhang, E. L. Reinherz, Enhancement to the RANKPEP resource for the prediction of peptide binding to MHC molecules using profiles. Immunogenetics 56, 405–419 (2004). 15349703 10.1007/s00251-004-0709-7

