# Supplementary material for: Immunogenicity and efficacy of the COVID-19 candidate vector vaccine MVA-SARS-2-S in preclinical vaccination
Source: Proc Natl Acad Sci U S A. 2021 Jun 23;118(28):e2026207118. doi: 10.1073/pnas.2026207118 (PMC8285915; doi:10.1073/pnas.2026207118)
Supplement: Supplementary File [file pnas.2026207118.sapp.pdf]

PNAS
www.pnas.org

Supplementary Information for

Immunogenicity and efficacy of the COVID-19 candidate vector vaccine MVA SARS 2 S in preclinical vaccination.

Alina Tscherne, Jan Hendrik Schwarz, Cornelius Rohde, Alexandra Kupke, Georgia Kalodimou, Leonard Limpinsel, Nisreen M.A. Okba, Berislav Bošnjak, Inga Sandrock, Ivan Odak, Sandro Halwe, Lucie Sauerhering, Katrin Brosinski, Nan Liangliang, Elke Duell, Sylvia Jany, Astrid Freudenstein, Jörg Schmidt, Anke Werner, Michelle Gellhorn Serra, Michael Klüver, Wolfgang Guggemos ,Michael Seilmaier, Clemens-Martin Wendtner, Reinhold Förster, Bart L. Haagmans, Stephan Becker, Gerd Sutter, Asisa Volz

Corresponding author: Gerd Sutter
Email: gerd.sutter@lmu.de

This PDF file includes:

Supplementary text
Figures S1 to S10
SI References

Supplementary Information Text

Materials and Methods.

Cell cultures. DF-1 cells (ATCC® CRL-12203™) were maintained in VP-SFM medium (Thermo Fisher Scientific, Planegg, Germany), 2% heat-inactivated fetal bovine serum (FBS) (Thermo Fisher Scientific, Planegg, Germany) and 2% L-glutamine (Thermo Fisher Scientific, Planegg, Germany). Primary chicken embryonic fibroblasts (CEF) were prepared from 10 to 11-day-old chicken embryos (SPF eggs, VALO, Cuxhaven, Germany) using recombinant trypsin (Tryple TM, Thermo Fisher Scientific, Planegg, Germany) and maintained in VP-SFM medium, 10% FBS and 1% L-glutamine. Vero cells (ATCC CCL-81) were maintained in Dulbecco`s Modified Eagle's Medium (DMEM), 10% FBS and 1% MEM non-essential amino acid solution (Sigma-Aldrich, Taufkirchen, Germany). Human A549 cells (ATCC® CCL-185™) (LGC standards) were maintained in DMEM with high glucose and 10% FBS. Human HeLa cells (ATCC CCL-2) were maintained in Minimum Essential Medium Eagle (MEM) (Sigma-Aldrich, Taufkirchen, Germany), 7% FBS and 1% MEM non-essential amino acid solution. Human HaCat cells (CLS Cell Lines Service, Eppelheim, Germany) were maintained in DMEM, 10% FBS, 1% MEM non-essential amino acid solution and 1% HEPES solution (Sigma-Aldrich, Taufkirchen, Germany). All cells were cultivated at 37 °C and 5 % CO_2.

Plasmid construction. The coding sequence of the full-length SARS-CoV-2 S protein (SARS-2-S) was modified *in silico* by introducing silent mutations to remove runs of guanines or cytosines and termination signals of vaccinia virus-specific early transcription. In addition, a C-terminal tag sequence encoding nine amino acids (YPYDVPDYA, aa 98-106 of influenza virus hemagglutinin, HAtag) (1) was added. The modified SARS-2-S cDNA was produced by DNA synthesis (Eurofins, Ebersberg, Germany) and cloned into the MVA transfer plasmid pIIIH5red under transcriptional control of the synthetic vaccinia virus early/late promoter PmH5 (2) to obtain the MVA expression plasmid pIIIH5red-SARS-2-S.

Generation of recombinant viruses. MVA vector viruses were obtained following the established protocols for vaccine development as described in previous studies (3-5). MVA (clonal isolate MVA-F6-sfMR) was grown on CEF under serum-free conditions and served a as non-recombinant backbone virus to construct MVA vector viruses expressing the SARS-CoV-2 S gene sequences. Briefly, monolayers of 90-95% confluent DF-1 or CEF cells were grown in six-well tissue culture plates (Sarstedt, Nürnbrecht, Germany), infected with non-recombinant MVA at 0.05 multiplicity of infection (MOI), and transfected with plasmid pIIIH5red-SARS-2-S DNA using X-tremeGENE HP DNA Transfection Reagent (Roche Diagnostics, Penzberg, Germany) according to the manual. Afterwards, cell cultures were collected and recombinant MVA viruses were clonally isolated by serial rounds of plaque purification on DF-1 or CEF cell monolayers monitoring for transient co-production of the red fluorescent marker protein mCherry. To obtain vaccine preparations, recombinant MVA–SARS-2-S were amplified on CEF or DF 1 cell monolayers grown in T175 tissue culture flasks, purified by ultracentrifugation through 36% sucrose and reconstituted to high titer stock preparations in Tris-buffered saline pH 7.4. Plaque-forming units (PFU) were counted to determine viral titers.

In vitro characterization of recombinant MVA-SARS-2-S. Genetic identity and genetic stability of vector viruses was confirmed by polymerase chain reaction (PCR) using viral DNA and detection of S-protein synthesis following serial passage at low MOI. For the latter, 95% confluent DF-1 cells were infected at MOI 0.05, incubated for 48h, harvested and used for reinfection. In total, five rounds of low MOI passage were performed. After the fifth passage, sixty virus isolates were obtained and amplified in 24-well DF-1 cultures for further testing. PCR analysis was performed to confirm genetic stability of viral genomes and MVA- and SARS-2-S-specific immunostaining served to monitor recombinant gene expression. The replicative capacity of recombinant MVA was tested in duplicate in multi-step-growth experiments on monolayers of DF-1, HaCat, HeLa or A549 cells grown in 6-well-tissue-culture plates. Viruses were inoculated at MOI 0.05, harvested at 0, 4, 8, 24, 48, and 72 h after infection, and titrated on CEF monolayers to determine infectivities in cell lysates in PFU.

Western Blot analysis of recombinant protein. To monitor production of the recombinant SARS-2-S protein, DF-1 cells were infected at MOI 10 with recombinant or non-recombinant MVA or remained uninfected (mock). At indicated time points of infection, cell lysates were prepared from infected cells and stored at −80 °C. Proteins from lysates were separated by electrophoresis in a sodium dodecyl sulfate (SDS)-10% polyacrylamide gel (SDS-PAGE; Bio-Rad, Munich) and subsequently transferred to a nitrocellulose membrane by electroblotting. The blots were blocked in a phosphate buffered saline (PBS) buffer containing 5% Bovine Serum Albumin (BSA) (Sigma-Aldrich, Taufkirchen, Germany) and 0.1% Tween-20 (Sigma-Aldrich, Taufkirchen, Germany) and incubated for 60 min with primary antibody, monoclonal anti-HAtag antibody (1:8000; HA Tag mAb 2-2.2.14, Thermo Fisher Scientific, Planegg, Germany) or COVID-19 patient serum (1:200). Next, membranes were washed with 0.1% Tween-20 in PBS and incubated with anti-mouse or anti-human IgG (1:5000; Agilent Dako, Glostrup, Denmark), conjugated to horseradish peroxidase. Blots were washed and developed using SuperSignal® West Dura Extended Duration substrate (Thermo Fisher Scientific, Planegg, Germany). Chemiluminescence was visualized using the ChemiDoc MP Imaging System (Bio-Rad, Munich, Germany). For use of patient serum ethical approval was granted by the Ethics Committee at the Medical Faculty of LMU Munich (vote 20-225 KB) in accordance with the guidelines of the Declaration of Helsinki.

Immunostaining of recombinant SARS-2-S protein. Vero cells were infected with 0.05 MOI MVA-SARS-2-S or non-recombinant MVA and incubated at 37 °C. After 24 h, cells were fixed with 4% paraformaldehyde (PFA) for 10 min on ice, washed two times with PBS, and permeabilized with 0.1% Triton X-100 (Sigma-Aldrich, Taufkirchen, Germany) solution in PBS. Permeabilized cells were probed with a monoclonal antibody against the HAtag epitope (1:1000; HAtag mAb 2-2.2.14, Thermo Fisher Scientific, Planegg, Germany) to detect SARS-2-S protein. Non-permeabilized cells were stained with a mouse monoclonal antibody obtained against the S protein of SARS-CoV-1 (SARS-1-S; 1:200; GeneTex) before fixation with PFA. Polyclonal goat anti-mouse secondary antibody (1:1000; Life Technologies, Darmstadt, Germany) was used to visualize S-specific staining by red fluorescence. Nuclei were stained with 1 µg/ml of 4,6-diamidino-2-phenylindole (DAPI) (Sigma-Aldrich, Taufkirchen, Germany) and cells were analyzed using the Keyence BZ-X700 microscope (Keyence, Neu-Isenburg, Germany) with a ×100 objective.

Vaccination experiments in mice. Female BALB/c mice (6 to 10 week-old) were purchased from Charles River Laboratories (Sulzfeld, Germany). Mice were maintained under specified pathogen-free conditions, had free access to food and water, and were allowed to adapt to the facilities for at least one week before vaccination experiments were performed. All animal

experiments were handled in compliance with the European and national regulations for animal experimentation (European Directive 2010/63/EU; Animal Welfare Acts in Germany). Immunizations were performed using intramuscular applications with vaccine suspension containing either 10^7 or 10^8 PFU recombinant MVA-SARS2-S, non-recombinant MVA or PBS (mock) into the quadriceps muscle of the left hind leg. Blood was collected on days 0, 18, or 35. Coagulated blood was centrifuged at 1300×g for 5 min in MiniCollect vials (Greiner Bio-One, Alphen aan den Rijn, The Netherlands) to separate serum, which was stored at −20 °C until further analysis.

Transduction of vaccinated mice with Ad_ACE2-mCherry and challenge infection with SARS-CoV-2. All animal experiments were performed in accordance with Animal Welfare Acts in Germany and were approved by the regional authorities. Vaccinated mice were housed under pathogen-free conditions and underwent intratracheal inoculation with $5x10^8$ PFU Adenovirus-ACE2-mCherry (cloned at ViraQuest Inc., North Liberty, IA, USA) under ketamine/xylazine anesthesia. Three days post transduction, mice were infected via the intranasal route with 1.5x104 tissue culture infectious dose 50 (TCID50) SARS-CoV-2 (BavPat1/2020 isolate, European Virus Archive Global # 026V-03883). Mice were sacrificed four days post infection and serum as well as lung tissue samples were taken for analysis of virus loads.

Quantitative real-time reverse transcription PCR to determine SARS-CoV-2 or mCherry RNA. Tissue samples of immunized and challenged mice were excised from the left lung lobes and homogenized in 1 ml DMEM. SARS-CoV-2 titres in supernatants (in TCID50 per ml) were determined on VeroE6 cells. RNA isolation was performed with the RNeasy minikit (Qiagen) according to the manufacturer's instructions. The RNA amount was measured using the NanoDrop ND-100 spectrophotometer. Total RNA was reverse transcribed and quantified by real-time PCR using the OneStep RT-PCR kit (Qiagen) as described previously (6) with the primer pair upE-Fwd and upE-Rev and the probe upE-Prb on a StepOne high-throughput fast real-time PCR system (ThermoFisher). Additionally, for every tissue sample from transduced and infected mice, evidence for successful ACE2 transduction was determined by real-time RT-PCR for mCherry mRNA with the OneStep RT-PCR kit (Qiagen). All samples for mCherry analysis were evaluated in one RT-PCR run. Quantification was carried out using a standard curve based on 10-fold serial dilutions of appropriate control RNA ranging from 10^2 to 10^5 copies.

Histopathological examination of lung tissue. Lungs were collected on day 4 post challenge with SARS-CoV-2 and processed for histological analysis. Briefly, tissue was fixed in formalin and embedded in paraffin. Four µm sections were cut with a microtome (RM2255, Leica Biosystems) and stained with hematoxylin and eosin (HE). To investigate the presence of viral RNA in lung tissue by in situ hybridization, the RNAscope® 2.5 HD Assay – RED Kit from Bio-Techne (Cat. No. 322360) was used according to the manufacturer’s instructions. Briefly, mounted slides were incubated at 60 °C, deparaffinized with xylene and 100% ethanol and pretreated with RNAscope® Pretreatment Reagents (Cat. No. 322300 and 322000) to enable access to the target RNA. Subsequently, a RNA-specific probe, targeted against the S gene of the SARS-CoV-2 (Cat. No. 848561), was hybridized to the RNA. Afterwards, signal amplification was performed and alkaline-phosphatase-labeled probes were used in combination with Fast Red substrate allowing signal detection. The slides were counterstained with Gill’s Hematoxylin I. A RNAscope® Negative Control Probe (Cat. No. 310043) was used in parallel to monitor background staining.

Antigen-specific IgG ELISA. SARS-2-S-specific serum IgG titres were measured by enzyme-linked immunosorbent assay (ELISA) as described previously (7). Flat bottom 96-well ELISA

plates (Nunc MaxiSorp Plates, Thermo Fisher Scientific, Planegg, Germany) were coated with 50 ng/well recombinant 2019-nCoV (COVID-19) S protein (Full Length-R683A-R685A-HisTag, ACROBiosystems, Newark, USA) overnight at 4 °C. Plates were washed and then blocked for 1 h at 37 °C with blocking buffer containing 1% BSA (Sigma-Aldrich, Taufkirchen, Germany) and 0.15M sucrose (Sigma-Aldrich, Taufkirchen, Germany) dissolved in PBS. Mouse sera were serially diluted three-fold down the plate in PBS containing 1% BSA (PBS/BSA), starting at a dilution of 1:100. Plates were then incubated for 1 h at 37 °C. After incubating and washing, plates were probed with 100 µl/well of goat anti-mouse IgG HRP (1:2000; Agilent Dako, Denmark) diluted in PBS/BSA for 1 h at 37 °C. After washing, 100 µl/well of 3´3´, 5´5´-Tetramethylbenzidine (TMB) Liquid Substrate System for ELISA (Sigma-Aldrich, Taufkirchen, Germany) was added until a colour change was observed. The reaction was stopped by adding 100µl/well of Stop Reagent for TMB Substrate (450 nm, Sigma-Aldrich, Taufkirchen, Germany). Absorbance was measured at 450 nm. The absorbance of each serum sample was measured at 450 nm with a 620 nm reference wavelength. ELISA data were normalized using the positive control. The cut-off value for positive mouse serum samples was determined by calculating the mean of the normalized OD 450nm values of the PBS control group sera plus 6 standard deviations (mean + 6 SD).

RBD-specific IgG ELISA. RBD-specific serum IgG titres were measured by enzyme-linked immunosorbent assay (ELISA). RBD ELISA was performed by coating 96-well microtitre plates with SARS-CoV-2 RBD protein in PBS overnight, as previously described (26). After blocking, serum dilutions (diluted 1:100) were incubated at 37°C for 1 h. Antigen-specific antibodies were detected by using peroxidase-labeled rabbit anti-human IgG (Dako) and 3,3′,5,5′-tetramethylbenzidine as a substrate. Absorbance was measured at 450 nm. A cutoff was set at an optical density of 0.5.

Surrogate virus neutralization assay (sVNT). To test for the presence of neutralizing anti-SARS-CoV-2-S serum antibodies we used surrogate virus neutralization test as described before with slight modifications (8). Briefly, 6 ng of SARS-CoV-2 S RBD (Trenzyme) was pre-incubated for 1 hour at 37 °C with heat-inactivated test sera at final dilutions between 1:20 to 1:540, as indicated on the graphs. Afterwards, SARS-CoV-2 S RBD-serum mixtures were loaded onto MaxiSorp 96F plates (Nunc) coated with 200 ng/well ACE2 [produced in-house as described in Bosnjak et al. (8)] and blocked with 2% bovine serum albumin/2% mouse serum (Invitrogen) and incubated for additional 1 h at 37 °C. As controls we used SARS-CoV-2-S-RBD pre-incubated only with buffer and non-specific mouse serum (Invitrogen). Plates were extensively washed with phosphate-buffered saline/0.05% Tween-20 (PBST), followed by incubation for 1 h at 37 °C with an HRP-conjugated anti-His-tag antibody (1.2 µg/ml; clone HIS 3D5). After appropriate washing, colorimetric signals were developed by addition of the chromogenic substrate 3,3',5,5'-tetramethylbenzidine (TMB; TMB Substrate Reagent Set, BD Biosciences) and stopped by addition of equal volume of 0.2 M H_2SO_4. The optical density values measured at 450 nm and 570 nm (SpectraMax iD3 microplate reader, Molecular Devices) were used to calculate percentage of inhibition after subtraction of background values as inhibition (%) = (1 - Sample OD value/Average SARS-CoV-2 S RBD OD value) x100. To remove background effects, the mean percentage of inhibition from non-specific mouse serum (Invitrogen) was deducted from sample values and neutralizing anti-SARS-CoV-2-S antibodies titres were determined as serum dilution that still had binding reduction > mean + 2 SD of values from sera of vehicle-treated mice.

Plaque reduction neutralization test 50 ($PRNT_{50}$). We tested serum samples for their neutralization capacity against SARS-CoV-2 (German isolate; GISAID ID EPI_ISL 406862;

European Virus Archive Global #026V-03883) by using a previously described protocol (9). We 2-fold serially diluted heat-inactivated samples in Dulbecco modified Eagle medium supplemented with NaHCO3, HEPES buffer, penicillin, streptomycin, and 1% foetal bovine serum, starting at a dilution of 1:10 in 50 µL. We then added 50 µL of virus suspension (400 plaque-forming units) to each well and incubated at 37°C for 1 h before placing the mixtures on VeroE6 cells (ATCC CRL1586). After incubation for 1 h, we washed, cells supplemented with medium, and incubated for 8 h. After incubation, we fixed the cells with 4% formaldehyde/phosphate-buffered saline (PBS) and stained the cells with polyclonal rabbit anti-SARS-CoV antibody (Sino Biological, https://www.sinobiological.com) and a secondary peroxidase-labeled goat anti-rabbit IgG (Dako, https://www.agilent.com). We developed the signal using a precipitate forming 3,3′,5,5′-tetramethylbenzidine substrate (True Blue; Kirkegaard and Perry Laboratories, https://www.seracare.com) and counted the number of infected cells per well by using an ImmunoSpot Image Analyzer (CTL Europe GmbH, https://www.immunospot.eu). The serum neutralization titre is the reciprocal of the highest dilution resulting in an infection reduction of >50% ($PRNT_{50}$). We considered a titre >20 to be positive.

SARS-CoV-2 virus neutralization test (VNT_{100}). The neutralizing activity of mouse serum antibodies was investigated based on a previously published protocol (10). Briefly, samples were serially diluted in 96-well plates starting from a 1:16 serum dilution. Samples were incubated for 1 h at 37°C together with 100 50% tissue culture infectious doses ($TCID_{50}$) of SARS-CoV-2 (BavPat1/2020 isolate, European Virus Archive Global # 026V-03883). Cytopathic effects (CPE) on VeroE6 cells (ATCC CRL1586) were analyzed 4 days after infection. Neutralization was defined as the absence of CPE compared to virus controls. For each test, a positive control (neutralizing COVID-19 patient plasma) was used in duplicates as an inter-assay neutralization standard. Ethical approval was granted by the Ethics Committee at the Medical Faculty of LMU Munich (vote 20-225 KB) in accordance with the guidelines of the Declaration of Helsinki.

Prediction and generation of synthetic SARS-2-S peptides. The sequence of the SARS-CoV-2 S protein (NCBI ID: QHD43416.1, Uniprot ID: P0DTC2 (SPIKE_SARS2)) served for epitope prediction, and probable CD8+ and CD4+ T cell determinants were examined with the Immune Epitope Database and Analysis Resource (IEDB, https://www.iedb.org/). For identification of potential CD8+ T cell determinants, the MHC-I Binding Prediction and MHC-I Processing Prediction tools (11, 12) were used and projections for 9-11mer peptides spanning the entire SARS-2-S protein sequence were obtained. The inputs selected for the search included the Prediction Method 'IEDB recommended 2.22', the MHC source species 'Mouse' and the MHC class I alleles $H2\text{-}K^d$, $H2\text{-}D^d$ and $H2\text{-}L^d$. The output was restricted to a percentile rank cut-off of 10.0. After lists of peptides were generated, all peptides with an IC50 score of 500nM or less were selected for inclusion in the top 5% list. All the peptides in this list were further analyzed using the MHC-I Processing Prediction tool 'Proteasomal cleavage/TAP transport/MHC class I combined predictor'. All peptides with an IC50 score of 500nM or less and a high total score were chosen and subsequently included in the top peptides list. To confirm that these peptides were potential binders of MHC class I alleles $H2\text{-}K^d$, $H2\text{-}D^d$ and $H2\text{-}L^d$, they were further screened for MHC I binding using the RankPep server (13). Peptides that were found to bind to any of the above alleles were selected for synthesis and testing.

For the identification of potential CD4+ T cell determinants, the MHC-II Binding Prediction tool (13) served to obtain 15mer peptides spanning the entire SARS-2-S protein sequence. The inputs for the analysis included the Prediction Method 'IEDB recommended 2.22', the MHC source species 'Mouse' and the MHC class II alleles $H2\text{-}IA^d$ and $H2\text{-}IE^d$. Peptides with percentile rank of 10.0 or less and an IC50 score of 1000 nM or less were further tested for MHC class II binding

using the RankPep server. Peptides bound to any of the above MHC class II alleles were selected for synthesis and testing. All peptides were obtained from Thermo Fisher Scientific (Planegg, Germany) as crude material (<50% purity) at a 1–4 mg scale, dissolved in PBS or DMSO to 2 mg/ml, aliquoted and stored at -20 °C.

Analysis of cellular response by Enzyme-Linked Immunospot (ELISPOT). At days 8 and 14 post prime or prime-boost vaccination, mice were sacrificed and splenocytes were prepared. Briefly, spleens were passed through a 70 µm strainer (Falcon®, Sigma-Aldrich, Taufkirchen, Germany) and incubated with Red Blood Cell Lysis Buffer (Sigma-Aldrich, Taufkirchen, Germany). Cells were washed and resuspended in RPMI-10 (RPMI 1640 medium containing 10% FBS, 1% Penicillin-Streptomycin, 1% HEPES; Sigma-Aldrich, Taufkirchen, Germany). ELISPOT assay (Mabtech ELISpot kit for mouse IFN-γ, Biozol, Eching, Germany) was performed to measure IFN-γ-producing T cells following the manufacturer's instructions. Briefly, $2x10^5$ splenocytes/100µl were seeded in 96-well plates and stimulated with individual peptides (2 µg/mL RPMI-10). Non-stimulated cells and cells stimulated with phorbol myristate acetate (PMA) / ionomycin (Sigma-Aldrich, Taufkirchen, Germany) or vaccinia virus peptide SPGAAGYD ($F2(G)_{26-34}$; $H\text{-}2L^d$;(14)) served as controls. After incubation at 37 °C for 48 h, plates were stained according to the manufacturer's instructions. Spots were counted and analyzed by using an automated ELISPOT plate reader and software following the manufacturer's instructions (A.EL.VIS Eli.Scan, A.EL.VIS ELISPOTAnalysis Software, Hannover, Germany).

T cell analysis by Intracellular Cytokine Staining (ICS). The detailed methods for intracellular cytokine staining (ICS) were described previously (7). Briefly, whole splenocytes were diluted in RPMI-10 and plated onto 96-well-U-bottom plates using 10^6 cells/well. Cells were stimulated with 8 µg/ml $S_{269-278}$ peptide or vaccinia virus peptide F_{226-34} for analysis of SARS-2-S- or MVA-specific CD8+ T cells. Splenocytes stimulated with PMA (10 ng/ml) plus ionomycin (500 ng/ml) served as positive controls and RPMI alone was used as a negative control. After 2 h at 37 °C, brefeldin A (Biolegend, San Diego, CA, USA) was added according to the manufacturer's instructions and stimulated cells were further maintained for 4 h at 37 °C. After the stimulations, cells were washed with FACS buffer (MACSQuant Running Buffer, Miltenyi Biotec, Bergisch Gladbach, Germany, plus 2% FBS) and stained extracellularly with anti-mouse CD3 phycoerithrin (PE)-Cy7 (clone 17A2, 1:100, Biolegend), anti-mouse CD4 Brilliant Violet 421 (clone GK1.5, 1:600, Biolegend), anti-mouse CD8α Alexa Fluor 488 (clone 53-6.8, 1:300, Biolegend), and purified CD16/CD32 (Fc block; clone 93, 1:500, Biolegend) using 50 µl/well diluted in FACS Buffer for 30 min on ice. After staining and washing, cells were incubated with 100 µl/well of the fixable dead cell viability dye Zombie Aqua (1:800, Biolegend) diluted in PBS for 30 min on ice. Cells were then washed, fixed with 100 µl/well of Fixation Buffer (Biolegend) for 20 min at room temperature, washed again, resuspended in 200 µl/well of FACS buffer and stored overnight at 4 °C. Next, cells were permeabilized using Intracellular Staining Permeabilization Wash Buffer (Perm Wash buffer; Biolegend; dilution 1:10), and stained intracellularly in 100 µl/well of anti-mouse IFN-γ (clone XMG1.2, 1:200, Biolegend) plus anti-mouse TNF-α (clone MP6-XT22, 1:200, Biolegend) diluted in Perm Wash buffer for 30 min at room temperature. Thereafter, cells were washed with Perm Wash buffer and resuspended in FACS buffer. Prior to analysis, samples were filtered through a 50 µm nylon mesh (Sefar Pty Ltd., Huntingwood, NSW, Australia) into 5 ml round bottom FACS tubes (Sarstedt, Nümbrecht, Germany). For each antibody, single colour controls were prepared using OneComp eBeads™ Compensation Beads (eBioscience, Thermo Fisher Scientific) and cells for the viability dye Zombie Aqua. Data was acquired by the MACSQuant VYB Flow Analyser (Miltenyi Biotec) and analyzed using FlowJo (FlowJo LLC, BD Life Sciences, Ashland, OR, USA).

Spectral flow cytometry. Spleens from immunized or mock-immunized groups of BALB/c mice (n=6) were obtained at day 14 after the last inoculation. Single cell suspensions of splenocytes in RPMI-10 were prepared by meshing the organs through 40 µm cell strainers. All samples were subjected to erythrocyte lysis. Non-specific antibody binding was blocked by incubating samples in 10% rat serum at 4 °C for 15 min. Without washing, cells were incubated with a mix of antibodies for additional 15 min at on 37 °C. The full list of antibodies and staining reagents is shown in Table S1. After washing, cells were acquired on Cytek Aurora spectral flow cytometer (Cytek) equipped with five lasers operating at 355 nm, 405 nm, 488 nm, 561 nm and 640 nm.

All flow cytometry data were analysed using FCS Express V7 (Denovo) and Graphpad Prism 7 (GraphPad). All samples were individually pre-gated (Fig. S8*A*) and subsequently concatenated into one file. The concatenated file was then pregated to exclude NK and NKT cells (Fig. S8*B*). Data processing included scaling, normalisation, weighted density downsampling to 200.000 cells and dimensionality reduction (UMAP). Heatmaps were used to identify cell clusters (Fig. S8*C*).

Table S1. Antibodies and staining reagents for spectral flow cytometry of mouse splenocytes.

Antigen	Clone	Fluorophore	Company	Cat. No.
CXCR3	CXCR3-173	BUV395	BD	745689
CD95	Jo2	BUV805	BD	741968
CCR6	29-2L17	BV 421	Biolegend	129818
CD4	GK1.5	BV 510	Biolegend	100449
CD62L	MEL-14	BV 570	Biolegend	104433
CD45.2	104	BV 605	Biolegend	109841
CD44	IM7	BV 650	Biolegend	103049
CD103	2E7	BV 711	Biolegend	121435
KLRG1	PK136	BV 750	BD	746876
CD3	17A2	AF532	Invitrogen	58-0032-82
CCR4	2G12	PerCP-Cy5.5	Biolegend	131220
CD49b	DX5	PE	BD	553858
CD69	H1.2F3	PE-Dazzle594	Biolegend	104536
CD127	SB/199	PE-Cy5	Biolegend	121124
Nrp1	3E12	PE-Cy7	Biolegend	145212
NKp46	29A1.4	eF660	eBioscience	50-3351-82
Viability	N/A	Zombie NIR	Biolegend	423106
CD8	53-6.7	APC-Cy7	Biolegend	100714

Statistical analysis. Data were prepared using GraphPad Prism version 5 (GraphPad Software Inc., San Diego CA, USA) and expressed as mean ± standard error of the mean (SEM). Data were analyzed by unpaired, two-tailed t-tests to compare two groups and one-way ANOVA to compare three or more groups. $P < 0.05$ was used as the threshold for statistical significance.

Supplementary Figures

Fig. S1. Molecular analysis of the MVA-SARS-2-S genome.

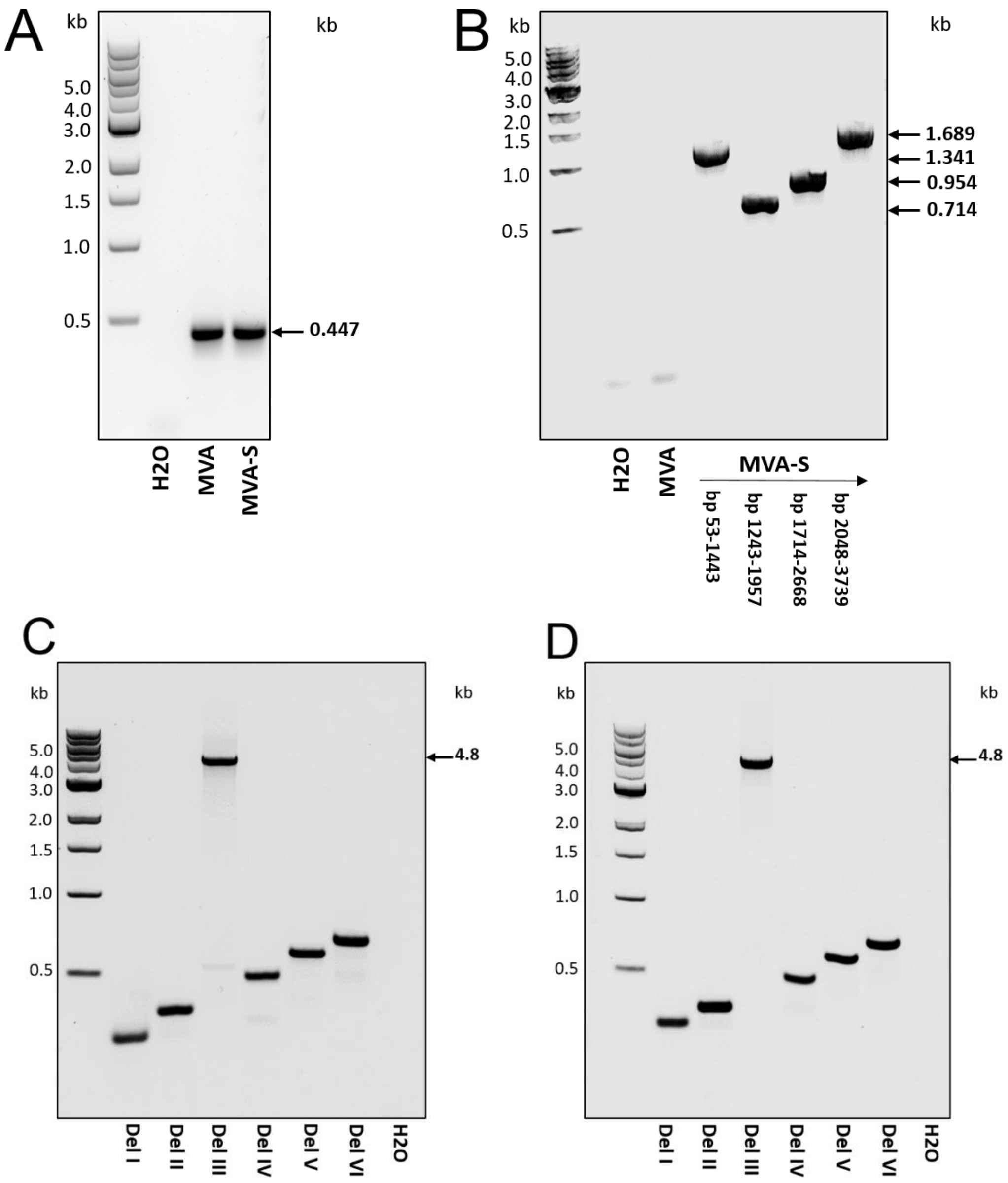

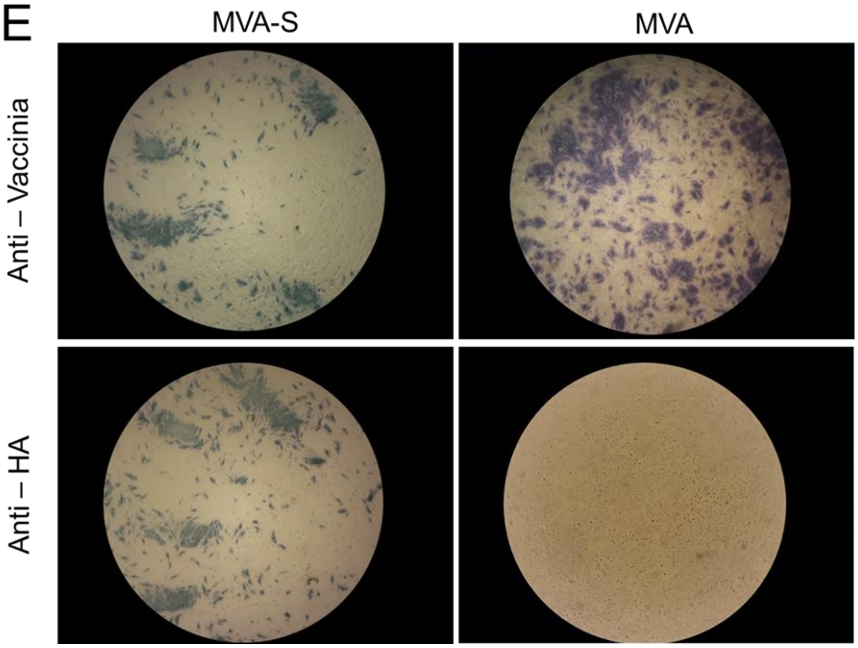

Fig. S1. PCR analysis of viral DNA to monitor (*A*) the *C7L* gene locus and (*B*) the SARS-CoV-2 S gene in the MVA-SARS-2-S (MVA-S) genome. (*A*) Amplification of a specific 447 bp DNA fragment from the MVA *C7L* gene sequence demonstrated integrity of the *C7L* gene locus in the MVA genome. The *C7L* gene is non-essential for MVA growth in chicken fibroblast cultures but the gene function is necessary to maintain unimpaired expression of MVA or recombinant genes under transcriptional control of vaccinia virus-specific late promoters (15). (*B*) Four different PCRs served to assess the integrity of the full-length SARS-2-S gene sequence inserted in the MVA-SARS-2-S genome. Amplified DNA fragments demonstrated the expected molecular weights with 1.341 kp (specific for S gene nucleotides 53-1443), 0.714 kb (nucleotides 1243-1957), 0.954 kb (nucleotides 1714-2668) and 1.689 kb (nucleotides 2048-3739). (*C*) Genetic stability of MVA-SARS-2-S after serial growth amplification in DF-1 cell cultures. DF-1 cells were infected with MVA-SARS-2-S at MOI of 0.05 and incubated for 48 h. Subsequently, the amplified virus was harvested and used to re-infect fresh DF-1 cells at MOI of 0.05 for 48 h. This procedure was performed five times. MVA-SARS-2-S genetic stability was tested by PCR analysis of genomic viral DNA and the monitoring for recombinant gene expression by S-specific immunostaining. PCR analysis demonstrated the genetic stability for six loci in the MVA-SARS-2-S genome (deletion sites Del I-VI) including the the heterologous SARS-CoV-2 S gene sequences inserted into the site of deletion III (Del III) with the amplification of characteristic size DNA fragments from viral DNA prepared after the first (*C*), or the fifth (*D*) round of MVA-SARS-2-S amplification in DF-1 cultures. (*E*) Following serial DF-1 passage MVA-SARS-2-S was tested by immunostaining for production of SARS-CoV-2 S protein using a mouse monoclonal antibody directed against the HAtag (anti-HA). A total of 60 clonal MVA-SARS-2-S (MVA-S) isolates were picked after the fifth virus amplification in DF-1 cells and used to infect fresh DF-1 cell monolayers. Infections with non-recombinant MVA (MVA) served as controls. After 48 h cell monolayers were fixed and stained with anti-vaccinia and anti-HA antibody. All MVA-SARS-2-S isolates (60/60) tested positive for unimpaired expression of recombinant S protein.

Fig. S2. MVA-SARS-2-S immunization schedules and safety monitoring**.**

A

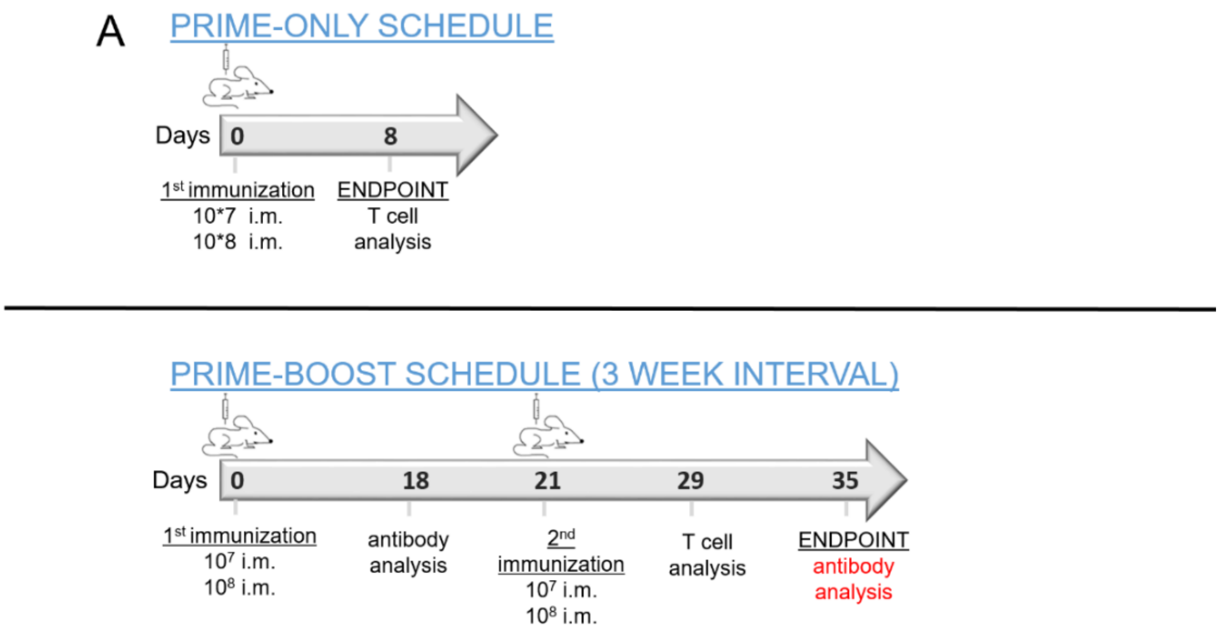

B

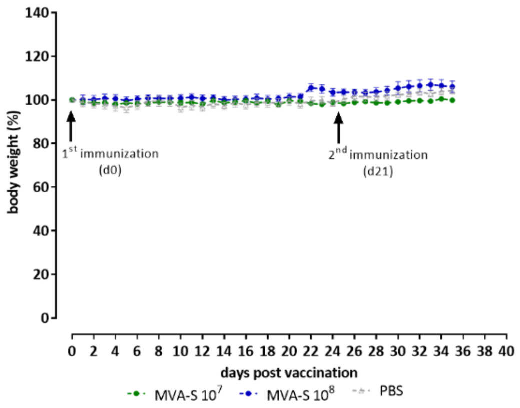

C

Main groups							Sex: female
Group		1			2		
Treatment		Vehicle			MVA-SARS-2-S prime boost		
Dose					10E8 pfu/animal		
Day of necropsy		35			35		
Grading		.	1	2	.	1	2
**Organs:**	**Microscopic findings:**						
Adrenal gland	No finding(s)	6/6	0/6	0/6	12/12	0/12	0/12
Kidney, L	No finding(s)	6/6	0/6	0/6	12/12	0/12	0/12
Liver	Inflammatory cells, multifocal	5/6	1/6	0/6	11/12	1/12	0/12
Lungs	Prominent BALT	6/6	0/6	0/6	11/12	1/12	0/12
Ln. subiliacus, L	Lymphoid hyperplasia	6/6	0/6	0/6	3/12	9/12	0/12
Ln. ischiadicus, L	Lymphoid hyperplasia	6/6	0/6	0/6	8/12	4/12	0/12
Ln. popliteus, L	Lymphoid hyperplasia	6/6	0/6	0/6	1/12	11/12	0/12
Ln. iliacus medialis, L	Lymphoid hyperplasia	6/6	0/6	0/6	1/12	11/12	0/12
Thymus	No finding(s)	6/6	0/6	0/6	12/12	0/12	0/12
Spleen	No finding(s)	6/6	0/6	0/6	12/12	0/12	0/12
Site of administration, L	Mixed cell infiltration, interstitial, multifocal	6/6	0/6	0/6	3/12	8/12	1/12
Site of administration, L	Necrosis, myofibres	6/6	0/6	0/6	10/12	2/12	0/12
Site of administration, L	Degeneration, myofibres	2/6	4/6	0/6	0/12	12/12	0/12

No. of animals affected / total No. of animals.

Fig. S2. MVA-SARS-2-S immunization schedules and monitoring for side effects of vaccination. Groups of BALB/c mice (n=6-12) were vaccinated with low dose 10^7 PFU (LD) or high dose 10^8 PFU (HD) MVA-SARS-2-S via the intramuscular (i.m.) route using two different schedules (prime-only n=6, prime-boost 3 week interval, n=12). (*A*) Schematic diagram of the immunization schedules. T cell responses were tested at day 8 after the 1st immunization (prime) or 2nd immunization (boost). Antibody responses (including S-binding and SARS-CoV-2 neutralizing antibodies) were examined three days before the 2nd immunization (d18) and 14 days post 2nd immunization (d35). (B) Monitoring for body weight changes of mice after prime-boost vaccination with MVA-SARS-2-S. Vaccination with saline (PBS) was used as a control. Body weights were measured daily. No signs of discomfort or disease were observed in vaccinated or control animals. (C) Histopathological examinations in prime-boost vaccinated animals. L = Left side. No lesions could be attributed to MVA-SARS-S-2 inoculation in any tissue other than the injection site and draining lymph nodes. Systemic effects related to vaccination were not seen. Signs of minimal to mild myodegeneration at the injection site were observed in treated and control mice.

Local inflammation of the myofiber interstitium and the adjacent adipose tissue was observed and interpreted as part of the physiological immune reaction to the vaccine virus as a consequence of the treatment procedure. The degree and extent of inflammation, myodegeneration and necrosis was in accordance with the ratio of inoculum volume in relation to the administration site. The lymphoid hyperplasia observed in draining lymph nodes is interpreted as a sign of immune competence of the animals and is characteristic for any early response to inflammation at a draining site. In conclusion, we observed no evidence for a potential toxicity of the full human dose of MVA-SARS-2-S in BALB/c mice. The repeated vaccination was well tolerated and caused no adverse events and no relevant macroscopic or histopathological changes. The observed reactions were comparable to previous experiments using non-recombinant MVA or other recombinant MVA vaccine constructs and are considered to be part of the pharmacodynamic principle of MVA-based vaccination (16).

Table S2. Selected SARS-CoV-2-S peptides with predicted MHC class I ($H2^d$) restriction

Peptide ID	Peptide	Length	Start	End	Pool #
S1	GYLQPRTFL	9	268	276	4
S2	AYSNNSIAI	9	706	714	10
S3	IYQAGSTPCNGV	12	472	483	5
S4	FTISVTTEI	9	718	726	10
S5	IYQTSNFRV	9	312	320	10
S6	IYQAGSTPC	9	472	480	5
S7	QYIKWPWYI	9	1208	1216	6
S8	CYGVSPTKL	9	379	387	11
S9	PPIKDFGGFNF	11	792	802	11
S10	VGYQPYRVVVL	11	503	513	7
S11	KYNENGTIT	9	278	286	4
S12	GYQPYRVVV	9	504	512	7
S13	QYGSFCTQL	9	755	763	8
S14	SYQTQTNSP	9	673	681	8
S15	YQPYRVVVL	9	505	513	7
S16	WPWYIWLGF	9	1212	1220	6
S17	VYAWNRKRI	9	350	358	9
S18	CGPKKSTNL	9	525	533	9
S19	KYFKNHTSP	9	1154	1162	9

Table S3. Selected SARS-CoV-2-S peptides with predicted MHC class II (IA^d and IE^d) restriction

.

Peptide ID	Peptide	Length	Start	End	Pool #
S20	TRFASVYAWNRKRIS	15	345	359	1
S21	RFASVYAWNRKRISN	15	346	360	1
S22	FASVYAWNRKRISNC	15	347	361	1
S23	INITRFQTLLALHRS	15	233	247	2
S24	NYLYRLFRKSNLKPF	15	450	464	2
S25	LIRAAEIRASANLAA	15	1012	1026	3
S26	NYNYLYRLFRKSNLK	15	448	462	2
S27	ASVYAWNRKRISNCV	15	348	362	1
S28	IRAAEIRASANLAAT	15	1013	1027	3
S29	GNYNYLYRLFRKSNL	15	447	461	2
S30	AAEIRASANLAATKM	15	1015	1029	3
S31	GGNYNYLYRLFRKSN	15	446	460	2
S32	RAAEIRASANLAATK	15	1014	1028	3
S33	ATRFASVYAWNRKRI	15	344	358	1
S34	NATRFASVYAWNRKR	15	343	357	1

Fig. S3. Comparison of SARS-CoV-2 neutralizing antibody responses.

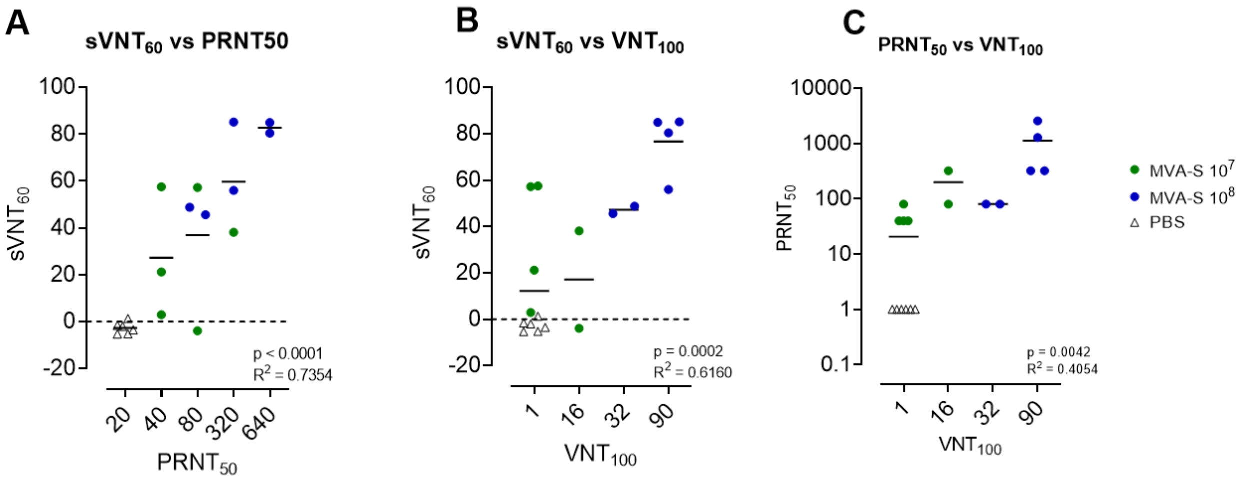

Fig. S3. Robust positive correlation of SARS-CoV-2 neutralizing antibody levels measured by $PRNT_{50}$, VNT_{100} and sVNT1:60. Correlation between (*A*) the percent inhibition of SARS-CoV-2 S RBD binding to ACE2 at a 1:60 serum dilution ($sVNT_{60}$) and $PRNT_{50}$ titers, (*B*) $sVNT_{60}$ and VNT_{100} titers; and (*C*) $PRNT_{50}$ and VNT_{100} titers. Correlation was done with one-way ANOVA followed by a test for the trend.

Fig. S4. Identification of H2-d restricted T cell epitopes of the SARS-CoV-2 S protein.

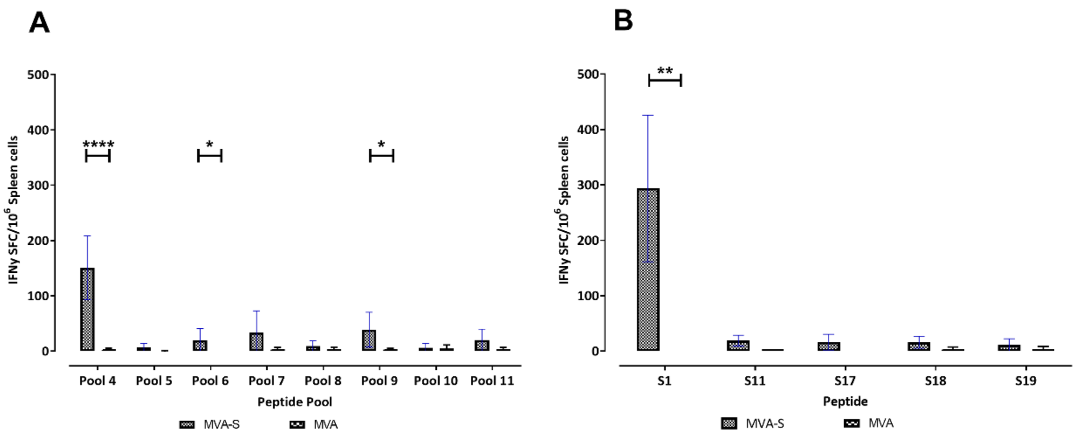

Fig. S4. Identification of H2-d restricted T cell epitopes of the SARS-CoV-2 S protein. Groups of BALB/c mice (n = 4 to 6) were immunized once with 10^8 PFU MVA-SARS-2-S (MVA-S) or non-recombinant MVA (MVA) via the i.m. route. Splenocytes were collected and prepared 8 days after the immunization. Total splenocytes were stimulated with pools of 9-12mer peptides or individual peptides from positive pools and were measured by IFN-y ELISPOT assays. (*A*) IFN-γ spot forming colonies (SFC) for stimulated splenocytes measured by ELISPOT assays after stimulation with peptide pools (2 to 3 peptides/pool). (*B*) IFN-γ SFC for stimulated splenocytes measured by ELISPOT assays after stimulation with each individual peptide from positive pools P4 and P9. Differences between MVA-S and MVA groups per peptide or peptide pool were analyzed by unpaired two-tailed t tests. Asterisks represent statistically significant differences between two groups: * $p < 0.05$; ** $p < 0.01$; **** $p < 0.0001$.

Fig. S5. Activation of MVA-specific CD8+ T cells after prime immunization.

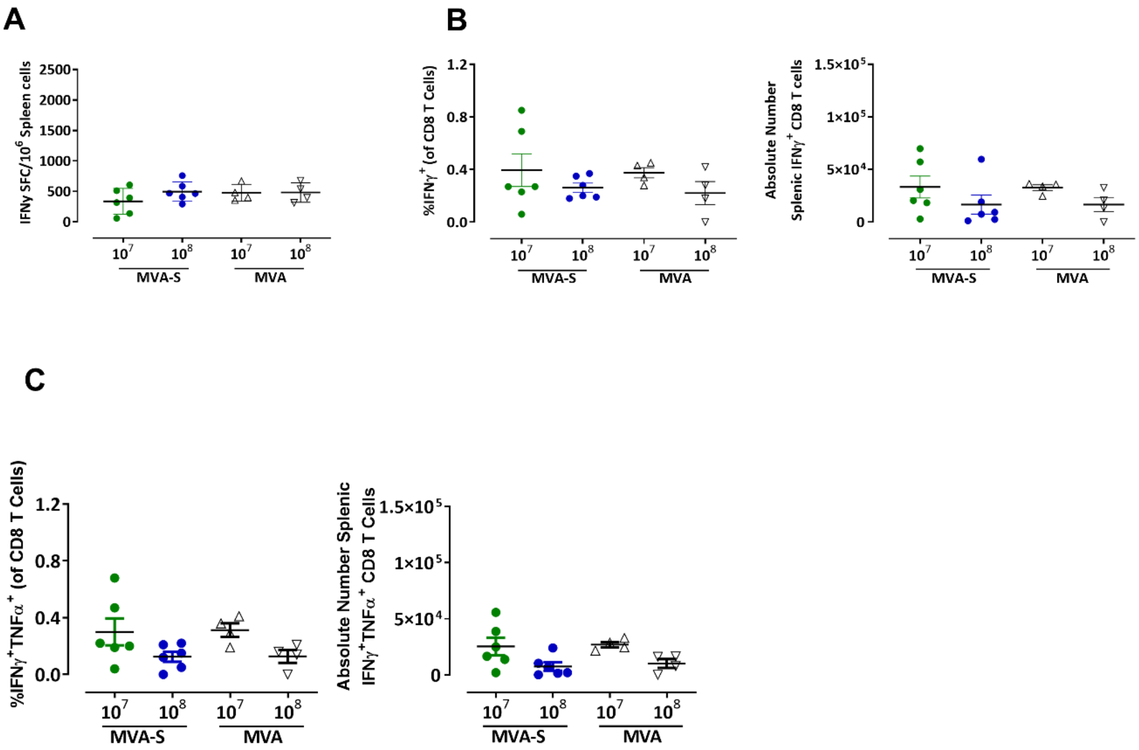

Fig. S5. Activation of MVA-specific CD8+ T cells after prime immunization with MVA-SARS-2-S. Groups of BALB/c mice (n = 4 to 6) were immunized once with 10^7 (LD) or 10^8 (HD) PFU MVA-SARS-2-S (MVA-S) or non-recombinant MVA (MVA) via the i.m. route. Splenocytes were collected and prepared 8 days after the immunization. Total splenocytes were stimulated with the H2d restricted MVA-specific peptide $F2_{26-34}$ and measured by IFN-γ ELISPOT assays and IFN-γ and TNF-α ICS plus FACS analysis. (*A*) IFN-γ spot forming colonies (SFC) for stimulated splenocytes measured by an ELISPOT assay. (*B*) IFN-γ production by CD8+ T cells measured by FACS analysis. Graphs show the frequency and absolute number of IFN-γ secreting CD8+ T cells. (*C*) IFN-γ and TNF-α production by CD8+ T cells measured by FACS analysis. Graphs show the frequency and absolute number of IFN-γ secreting TNF-α+ CD8+ T cells.

Fig. S6. Activation of MVA-specific CD8+ T cells after prime-boost immunization.

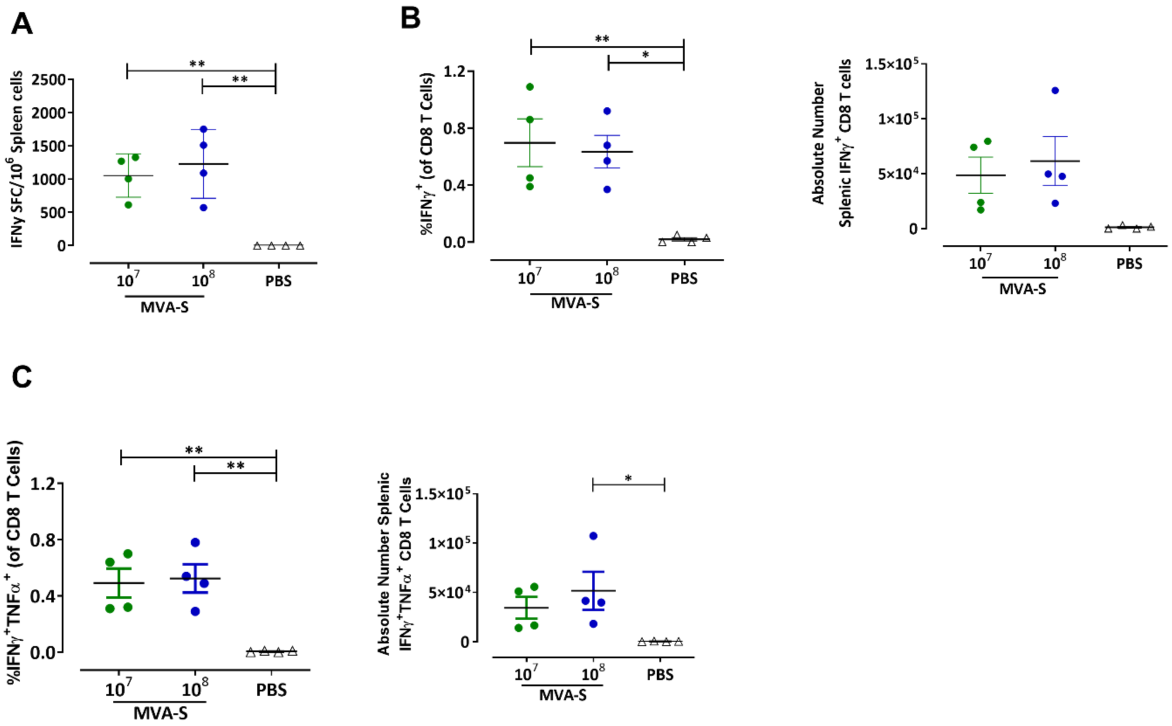

Fig. S6. Activation of MVA-specific CD8+ T cells after prime-boost immunization (21-day interval) with MVA-SARS-2-S. Groups of BALB/c mice (n = 4) were immunized twice with 10^7 (LD) or 10^8 (HD) PFU MVA-SARS-2-S (MVA-S) or non-recombinant MVA (MVA) over a 21-day interval via the i.m. route. Splenocytes were collected and prepared 8 days after the final immunization. Total splenocytes were stimulated with the H2d restricted MVA-specific peptide $F2_{26-34}$ and were measured by IFN-γ ELISPOT assays and IFN-γ and TNF-α ICS plus FACS analysis. (*A*) IFN-γ SFC for stimulated splenocytes measured by ELISPOT assay. (*B*) IFN-γ production by CD8+ T cells measured by FACS analysis. Graphs show the frequency and absolute number of IFN-γ+ CD8+ T cells. (*C*) IFN-γ and TNF-α production by CD8+ T cells measured by FACS analysis. Graphs show the frequency and absolute number of IFN-γ+ TNF-α+ CD8+ T cells. Differences between groups were analyzed by one-way ANOVA and Tukey post-hoc test. Asterisks represent statistically significant differences between two groups. * $p < 0.05$, ** $p < 0.01$.

Fig. S7. Representative flow cytometry plots of the CD8+ T cell compartment.

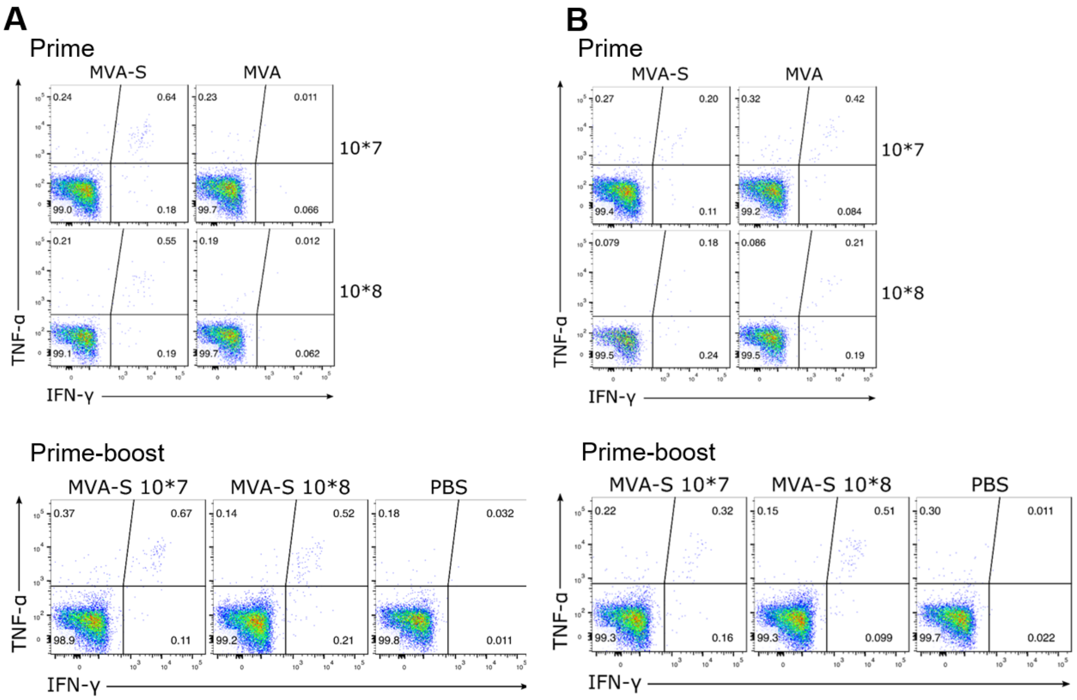

Fig. S7. Representative flow cytometry plots of the IFN-γ+ versus TNF-α+ CD8+ T cell compartment after prime and prime-boost vaccination. Groups of BALB/c mice (n = 4 to 6) were immunized once or twice with 10^7 (LD) or 10^8 (HD) PFU MVA-SARS-2-S (MVA-S) or non-recombinant MVA (MVA) or saline (PBS) via the i.m. route. Splenocytes were collected and prepared 8 days after the last immunization. Total splenocytes were stimulated either (*A*) with the H2d-restricted peptide $S_{268-276}$ (S1; GYLQPRTFL) or (*B*) with the H2d-restricted MVA-specific peptide $F2_{26-34}$ and measured by IFN-γ and TNF-α ICS plus FACS analysis.

Fig. S8. Spectral flow cytometry of T cell subsets.

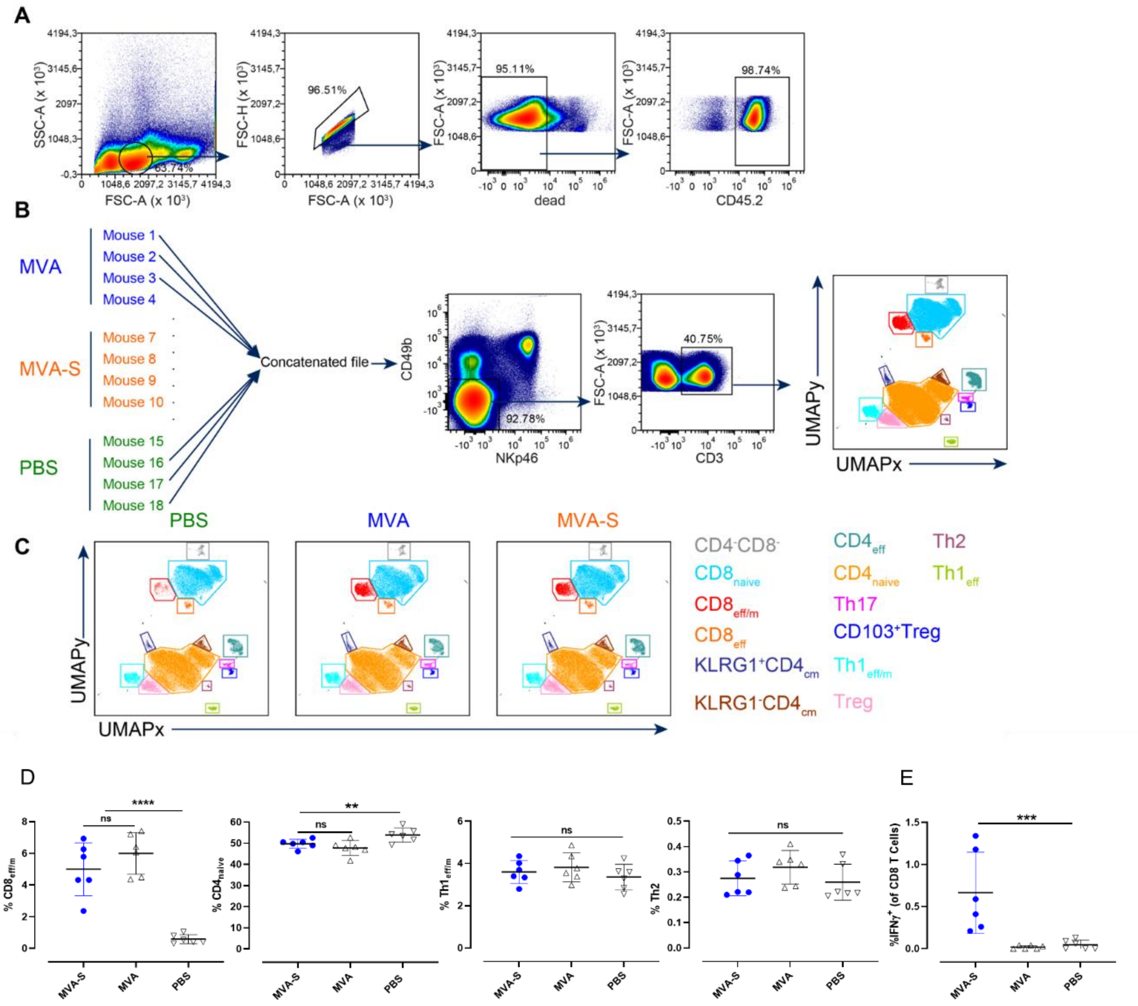

Fig. S8. Spectral flow cytometry of splenocytes after prime-boost immunization with MVA-SARS-2-S. Groups of BALB/c mice (n=6) were immunized twice with 10^8 (HD) PFU MVA-SARS-2-S (MVA-S), non-recombinant MVA (MVA) or saline (PBS) via the i.m. route. Spleens were collected and single cell suspensions prepared 14 days after the last immunization. Splenocytes were incubated with a mix of antibodies to stain T cell markers (see Table S1). After washing, cells were acquired on a Cytek Aurora spectral flow cytometer. (*A*) All samples were individually pre-gated and subsequently concatenated into one file. (*B*) The concatenated file was then gated to exclude NK and NKT cells. (*C*) Heatmaps were used to identify cell clusters. (*D*) Data from selected T cell subsets show high levels of CD8+ effector memory T cells (CD8eff/m) and reduced levels of naïve CD4+ T cells ($CD4_{naive}$) in MVA-S and MVA immunized animals, whereas, comparable populations of T helper cell populations were detected. (*E*) IFN-γ production measured by ICS and conventional FACS analysis. Stimulation with SARS-CoV-2-specific peptide $S_{268-276}$ (S1; GYLQPRTFL) shows the specific detection of IFN-γ+ CD8+ T cells in splenocytes from MVA-SARS-2-S immunized mice. Differences between groups were analyzed by one-way ANOVA and Tukey post-hoc test. Asterisks represent statistically significant differences between two groups. *** $p < 0.001$, **** $p < 0.0001$.

Fig. S9. ELISPOT to monitor activation of CD4+ T cells.

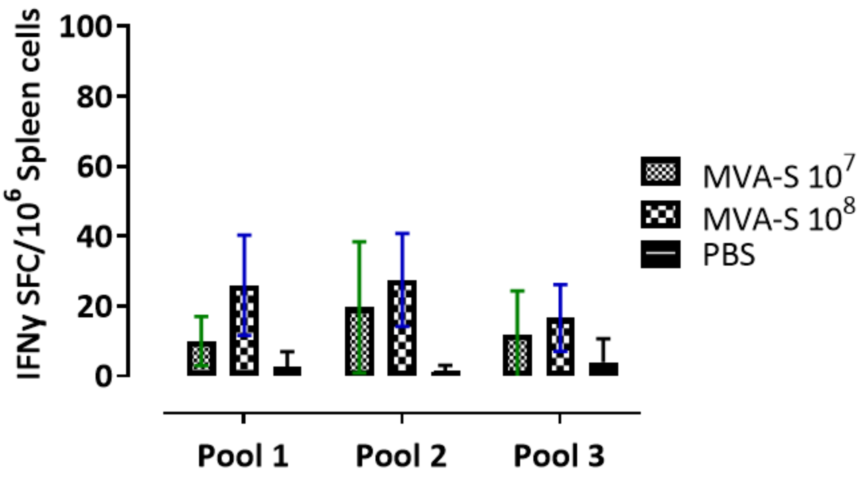

Fig. S9. ELISPOT to monitor activation of CD4+ T cells in BALB/c mice. Groups of BALB/c mice (n=4 to 6) were i.m. immunized in a prime-boost regime (21-day interval) with 10^8 and 10^7 PFU of MVA-SARS-2-S (MVA-S). Mice vaccinated with saline (PBS) were used as a controls. Splenocytes were collected at day 8 post 2nd immunization and stimulated with pools (4 to 6 peptides /pool) of 15mer SARS-2-S derived peptides (Table S2). IFN-γ spot-forming cells were counted by ELISPOT.

Fig. S10. Clinical outcome of SARS-CoV-2 infection**.**

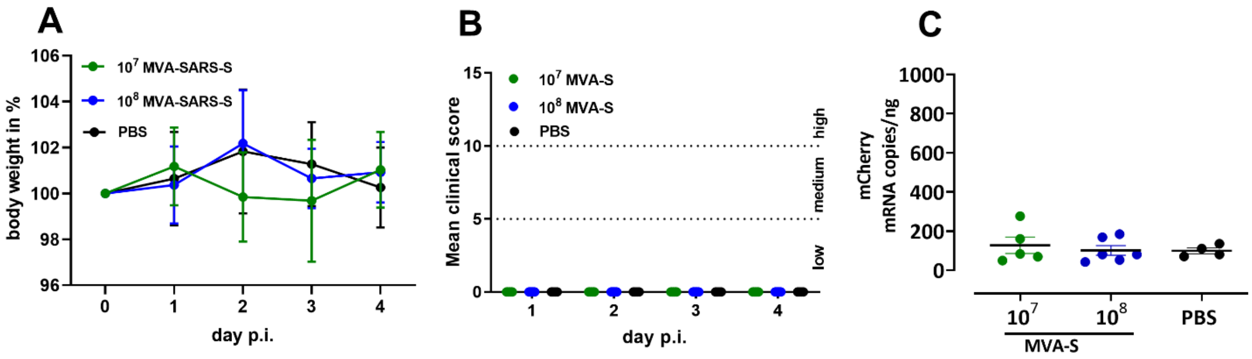

Fig. S10. Clinical outcome of SARS-CoV-2 infection in hACE2-transduced and MVA-SARS-2-vaccinated mice. Groups of BALB/c mice (n = 4-6) were i.m. immunized twice with 10^7 or 10^8 PFU of MVA-SARS-2-S (MVA-S) over a 21-day interval. Mock immunized mice (PBS) served as controls. About two weeks after the last immunization the mice were sensitized with an adenovirus expressing hACE2 and mCherry and infected with SARS-CoV-2 three days after transduction. (*A*) Body weight change was monitored daily, (*B*) spontaneous behavior, and general condition which were summarized in a clinical score, (*C*) four days after SARS-CoV-2 infection mice were sacrificed, lungs were isolated and mCherry mRNA copies were evaluated.

SI References

1. I. A. Wilson *et al.*, The structure of an antigenic determinant in a protein. *Cell* **37**, 767-778 (1984).
2. L. S. Wyatt, S. T. Shors, B. R. Murphy, B. Moss, Development of a replication-deficient recombinant vaccinia virus vaccine effective against parainfluenza virus 3 infection in an animal model. *Vaccine* **14**, 1451-1458 (1996).
3. T. Koch *et al.*, Safety and immunogenicity of a modified vaccinia virus Ankara vector vaccine candidate for Middle East respiratory syndrome: an open-label, phase 1 trial. *Lancet Infect. Dis.* **20**, 827-838 (2020).
4. J. H. C. M. Kreijtz *et al.*, Safety and immunogenicity of a modified-vaccinia-virus-Ankara-based influenza A H5N1 vaccine: a randomised, double-blind phase 1/2a clinical trial. *The Lancet Infect. Dis.* **14**, 1196-1207 (2014).
5. F. Song *et al.*, Middle East respiratory syndrome coronavirus spike protein delivered by modified vaccinia virus Ankara efficiently induces virus-neutralizing antibodies. *J. Virol.* **87**, 11950-11954 (2013).
6. V. M. Corman *et al.*, Detection of 2019 novel coronavirus (2019-nCoV) by real-time RT-PCR. *Euro Surveill.* **25** (2020).
7. G. Kalodimou *et al.*, A Soluble Version of Nipah Virus Glycoprotein G Delivered by Vaccinia Virus MVA Activates Specific CD8 and CD4 T Cells in Mice. *Viruses* **12** (2019).
8. B. Bošnjak *et al.*, Low serum neutralizing anti-SARS-CoV-2 S antibody levels in mildly affected COVID-19 convalescent patients revealed by two different detection methods. *Cell. Mol. Immunol.* 10.1038/s41423-020-00573-9 (2020).
9. N. M. A. Okba *et al.*, Severe Acute Respiratory Syndrome Coronavirus 2−Specific Antibody Responses in Coronavirus Disease Patients. *Emerg Infect. Dis. 26(7):1478-1488* **26** (2020).
10. C. Kreer *et al.*, Longitudinal Isolation of Potent Near-Germline SARS-CoV-2-Neutralizing Antibodies from COVID-19 Patients. *Cell* **182**, 843-854.e812 (2020).
11. S. K. Dhanda *et al.*, IEDB-AR: immune epitope database-analysis resource in 2019. *Nucleic Acids Res.* **47**, W502-w506 (2019).
12. W. Fleri *et al.*, The Immune Epitope Database and Analysis Resource in Epitope Discovery and Synthetic Vaccine Design. *Front. Immunol.* **8** (2017).

13. P. A. Reche, J.-P. Glutting, H. Zhang, E. L. Reinherz, Enhancement to the RANKPEP resource for the prediction of peptide binding to MHC molecules using profiles. *Immunogenetics* **56**, 405-419 (2004).
14. D. C. Tscharke *et al.*, Poxvirus CD8+ T-Cell Determinants and Cross-Reactivity in BALB/c Mice. *J. Virol.* **80**, 6318-6323 (2006).
15. S. Backes *et al.*, Viral host-range factor C7 or K1 is essential for modified vaccinia virus Ankara late gene expression in human and murine cells, irrespective of their capacity to inhibit protein kinase R-mediated phosphorylation of eukaryotic translation initiation factor 2alpha. *J. Gen. Virol.* **91**, 470-482 (2010).
16. M. C. Langenmayer *et al.*, Distribution and absence of generalized lesions in mice following single dose intramuscular inoculation of the vaccine candidate MVA-MERS-S. *Biologicals* **54**, 58-62 (2018).
